# Highly Emissive 9‐Borafluorene Derivatives: Synthesis, Photophysical Properties and Device Fabrication

**DOI:** 10.1002/chem.202005185

**Published:** 2021-03-10

**Authors:** Xing Chen, Guoyun Meng, Guanming Liao, Florian Rauch, Jiang He, Alexandra Friedrich, Todd B. Marder, Nan Wang, Pangkuan Chen, Suning Wang, Xiaodong Yin

**Affiliations:** ^1^ Key Laboratory of Cluster Science Ministry of Education of China Beijing Key Laboratory of Photoelectronic/Electrophotonic, Conversion Materials School of Chemistry and Chemical Engineering Beijing Institute of Technology 102488 Beijing P.R. China; ^2^ Institut für Anorganische Chemie Institute for Sustainable Chemistry & Catalysis with Boron (ICB) Julius-Maximilians-Universität Würzburg Am Hubland 97074 Würzburg Germany; ^3^ Department of Chemistry Queen's University Kingston ON K7L3N6 Canada

**Keywords:** boron heterocycles, density functional calculations, luminescence, organic light-emitting diodes, photophysics

## Abstract

A series of 9‐borafluorene derivatives, functionalised with electron‐donating groups, have been prepared. Some of these 9‐borafluorene compounds exhibit strong yellowish emission in solution and in the solid state with relatively high quantum yields (up to 73.6 % for **FMesB‐Cz** as a neat film). The results suggest that the highly twisted donor groups suppress charge transfer, but the intrinsic photophysical properties of the 9‐borafluorene systems remain. The new compounds showed enhanced stability towards the atmosphere, and exhibited excellent thermal stability, revealing their potential for application in materials science. Organic light‐emitting diode (OLED) devices were fabricated with two of the highly emissive compounds, and they exhibited strong yellow‐greenish electroluminescence, with a maximum luminance intensity of >22 000 cd m^−2^. These are the first two examples of 9‐borafluorene derivatives being used as light‐emitting materials in OLED devices, and they have enabled us to achieve a balance between maintaining their intrinsic properties while improving their stability.

## Graphical abstract

**Luminescent boroles**: Highly twisted donor‐functionalised 9‐borafluorenes have been synthesised that exhibit bright luminescence without obvious charge‐transfer character, as well as excellent thermal stability, suggesting their suitability for optoelectronic applications (see figure). Two organic light‐emitting diode devices fabricated with these compounds showed properties highly favourable for future development.

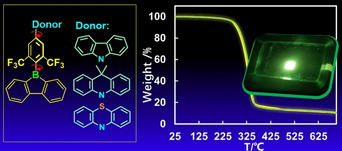

## Introduction

Three‐coordinate boron‐containing materials have attracted considerable attention over the last several decades due to the conjugation between the vacant p orbital on boron and the π electrons of conjugated systems. This conjugation leads to desirable optical and electronic properties that, in turn, enable applications in optoelectronics and sensing materials.[^1^, ^2^, ^3^, ^4^, ^5^, ^6^, ^7^, ^8^, ^9^, ^10^, ^11^, ^12^, ^13^, ^14^, ^15^] For example, several groups have shown that three‐coordinate boron, as an electron‐acceptor, combined with electron‐donor groups, giving D–A systems, can exhibit thermally activated delayed fluorescence (TADF), which is favourable for organic light‐emitting diode (OLED) devices.[^16^, ^17^, ^18^, ^19^, ^20^, ^21^, ^22^, ^23^, ^24^, ^25^, ^26^, ^27^, ^28^, ^29^] Among three‐coordinate boranes, boroles are unique because of their 4π‐electron five‐membered‐ring structure, which presents antiaromatic character, according to Hückel's rule. Due to their antiaromaticity, boroles exhibit high Lewis acidity, and thus instability towards ambient conditions (i.e., air and water),^[30]^ unless the vacant orbital on boron is sterically protected. The first borole, pentaphenylborole, was reported half a century ago by Eisch et al.;^[31]^ however, its crystal structure was determined only a little over 10 years ago.[^32^, ^33^] Recently, Marder and co‐workers used a very bulky and highly electron‐deficient 1,3,5‐tris(trifluoromethyl)benzene (^F^Mes) group to stabilise boroles. Interestingly, the stability of these ^F^Mes‐protected boroles was improved significantly and, at the same time, their low reduction potentials and pronounced antiaromaticity were maintained.^[34]^ Dibenzoboroles, also widely known as 9‐borafluorenes, exhibit significantly enhanced stability because of their reduced antiaromatic character, due to the delocalisation of π electrons over the fused biphenylene backbone.[^35^, ^36^] Benefiting from their easy accessibility, 9‐borafluorenes have been explored as a platform for chemical sensors,^[37]^ novel ring‐extension reactions[^38^, ^39^, ^40^, ^41^, ^42^] and small‐molecule activation.^[43]^ However, their enhanced stability typically comes at the expense of their acceptor properties, as the LUMO is typically much higher in energy. Thus, the challenge is to retain the low‐lying LUMO of a simple borole, while greatly enhancing the stability of the system.

**Figure 1:**
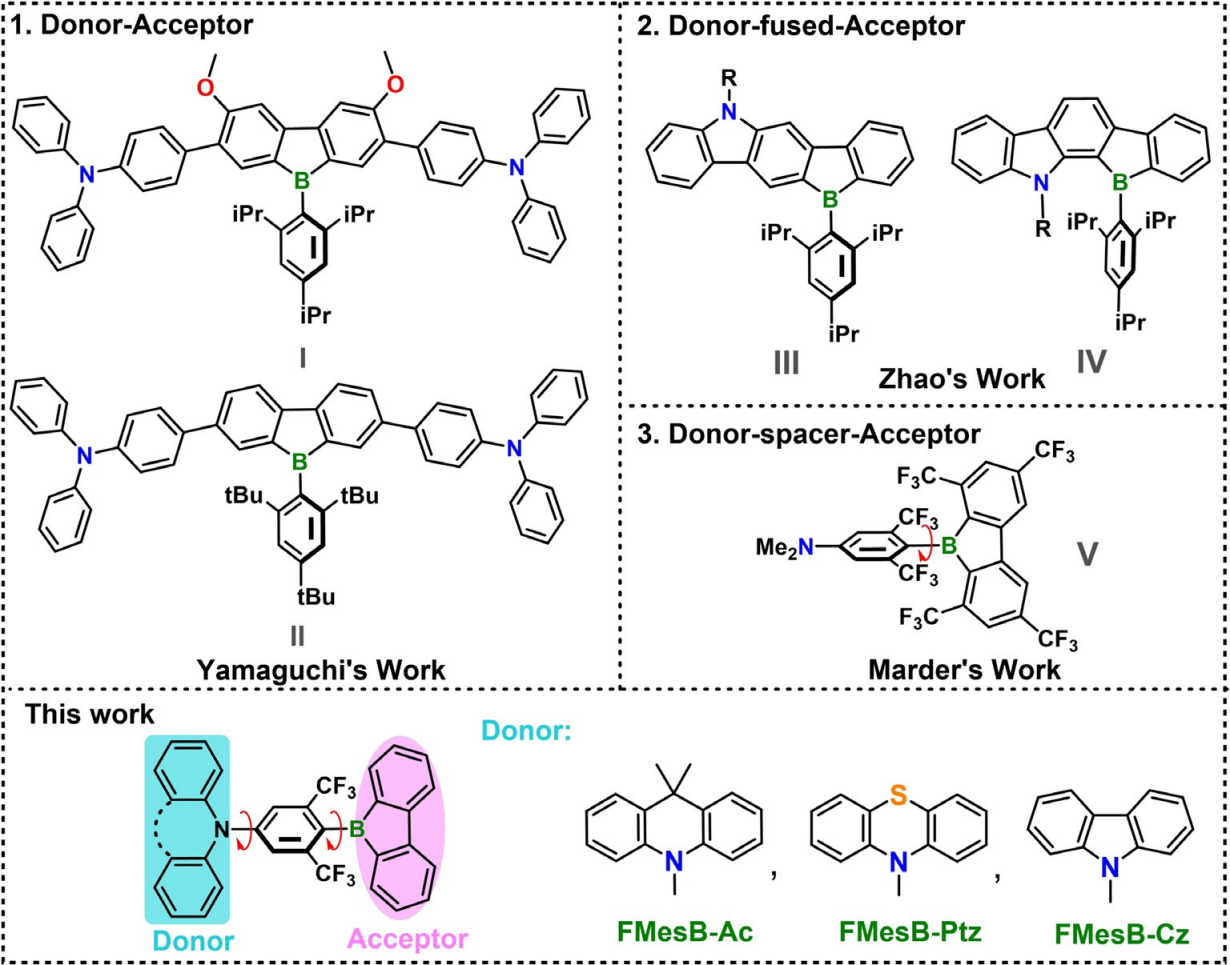
Previously reported borafluorenes and those discussed in the current work.

To explore the potential applications of 9‐borafluorenes in functional materials, multiple D–A systems with 9‐borafluorene as the acceptor unit and an amine as the donor have been studied (Figure 1). Yamaguchi and co‐workers observed strong solvatochromic behaviour of both the absorption and emission of **I** and **II** (Figure 1), which suggests a high degree of charge‐transfer character and strong Lewis acidity.[^44^, ^45^] In contrast, Zhao and co‐workers observed only limited solvatochromism in both the absorption and emission of **III** and **IV**, which indicates localised excitation character (Figure 1).^[46]^ This could be due to highly efficient electron delocalisation in the ground state, as a result of the rigid ladder framework. More recently, Marder and co‐workers reported a strategy to enhance the π‐accepting ability (i.e., low‐lying LUMO) of borafluorenes while maintaining their stability, that is, to overcome the stability–property trade‐off that is normally encountered in 9‐borafluorenes.^[47]^ They synthesised a trifluoromethylated borafluorene with a dimethylamino group on the *exo*‐aryl moiety at the position *para* to boron, namely *p*‐NMe_2_‐^F^Xyl^F^Bf (**V**; Figure 1), which exhibits a reduction potential of around −1.28 V (vs. Fc/Fc^+^), and the twisted D–A structure results in TADF with a delayed fluorescence lifetime of around 1.6 μs. However, its low quantum yield (ca. 0.03) limits its application in optical materials. The above reports show that D–A systems with borafluorene acceptors often exhibit interesting photophysical and electrochemical properties, which greatly widens the potential applications of borafluorene derivatives in chemistry and materials.

In this study, we expanded upon this strategy for enhancing the chemical and thermal stability of 9‐borafluorenes by introducing nitrogen‐based electron donors of varying donor strength at the *para* position of the *exo*‐aryl moiety of 9‐borafluorenes. The subtle changes in the electronic structure provide insight into the fundamental properties of borafluorene D–A systems. As *o*‐trifluoromethyl groups have proven to be excellent protecting groups for borafluorenes in previous work, bis(trifluoromethyl)phenylene was selected as the spacer.[^48^, ^49^, ^50^] Unsubstituted 9‐borafluorene was chosen as acceptor, considering the ease of synthetic access and comparability with other 9‐borafluorene derivatives.

## Results and Discussions

### Synthesis of donor‐functionalised borafluorenes

**Figure 2:**
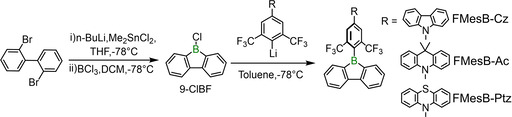
Synthetic route to **FMesB‐Ac**, **FMesB‐Ptz** and **FMesB‐Cz**.

The borafluorene precursor **9‐ClBF** was prepared according to the literature (Figure 2),^[49]^ and the donor‐functionalised bis(trifluoromethyl)benzene compounds were synthesised by Buchwald–Hartwig coupling reactions[^51^, ^52^, ^53^] (see the Supporting Information). The donor‐functionalised bis(trifluoromethyl)benzene was treated with *n*BuLi in Et_2_O at −78 °C for 30 min, then warmed to room temperature and stirred for another 3 hours to give the corresponding lithiated donor‐functionalised bis(trifluoromethyl)benzene. The lithiated species were subsequently mixed with **9‐ClBF** in dry toluene at −78 °C, and then warmed to room temperature to give **FMesB‐Cz**, **FMesB‐Ac** and **FMesB‐Ptz** in overall yields of 23, 29 and 29 %, respectively (Figure 2). These compounds are light‐yellow powders and are stable in air. They were fully characterised by NMR spectroscopy and HRMS (see the Supporting Information).

### Molecular structures of the donor‐functionalised 9‐borafluorenes

**Figure 3:**
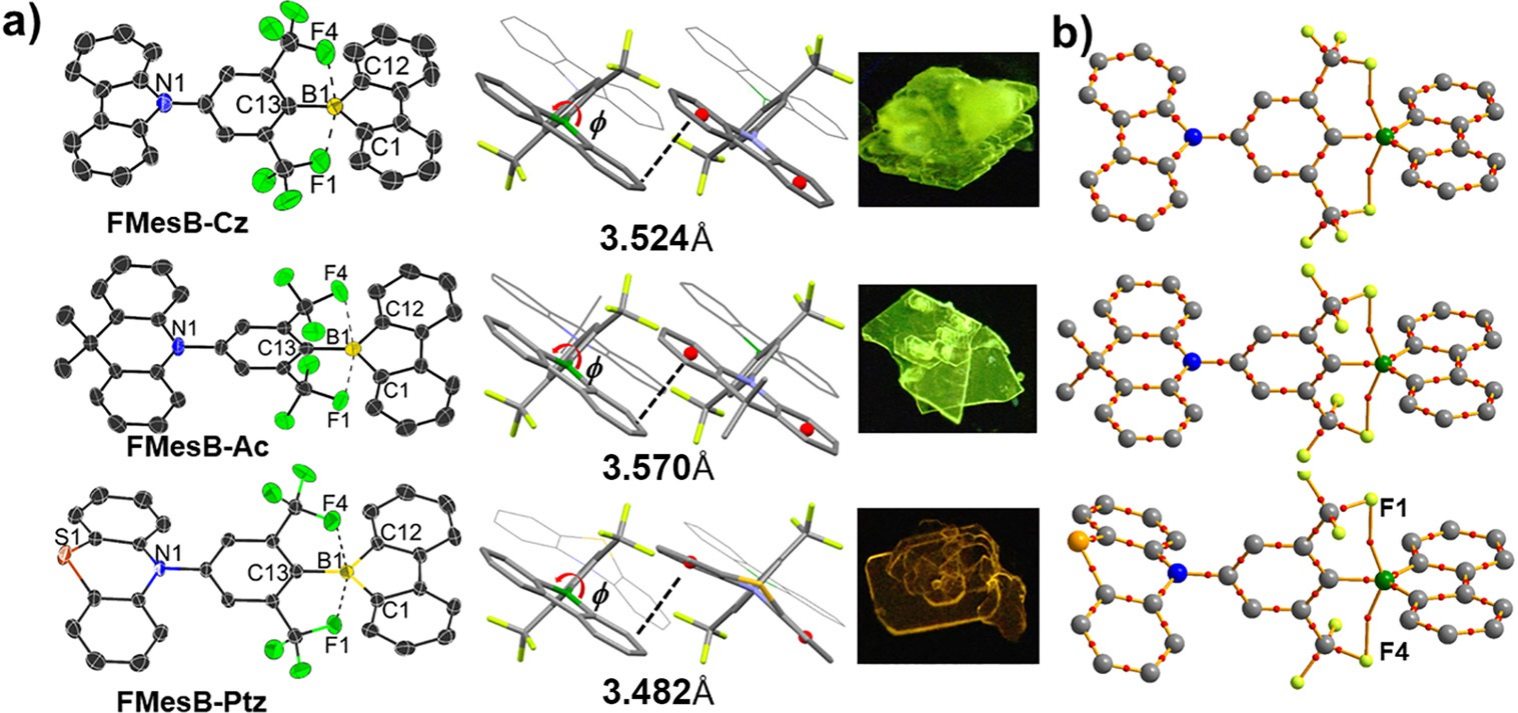
(a) Molecular structures determined by single‐crystal XRD analysis at 298 K for **FMesB‐Cz** and at 180 K for **FMesB‐Ac** and **FMesB‐Ptz**. Thermal ellipsoids are drawn at the 50 % probability level. Hydrogen atoms have been omitted for clarity. Selected bond lengths [Å] for **FMesB‐Cz**: B1−C1 1.562(3), B1−C12 1.567(3), B1−C13 1.580(3); for **FMesB‐Ac**: B1−C1 1.553(3), B1−C12 1.560(3), B1−C13 1.584(3); for **FMesB‐Ptz**: B1−C1 1.562(2), B1−C12 1.561(2), B1−C13 1.581(2). The pictures show the crystals under a UV lamp. (b) Optimised geometries and AIM analysis of **FMesB‐Cz**, **FMesB‐Ac** and **FMesB‐Ptz**, showing the B⋅⋅⋅F bond paths (purple lines) and BCPs (red points).

Single crystals of the three compounds were obtained by recrystallisation from hexane, and the structures are shown in Figure 3. The bond lengths between the boron and the neighbouring carbon atoms are similar to those of the ^F^Mes‐capped 9‐borafluorene (^**F**^ **MesBf**).^[49]^ The bis(trifluoromethyl)phenyl groups adopt orientations almost orthogonal to the planes of the borafluorene moiety in all of the compounds, with torsion angles of 75.5(2) and 77.0(2)° for **FMesB‐Cz**, 83.5(1)° for **FMesB‐Ac** and 82.8(1)° for **FMesB‐Ptz**. All of the dihedral angles are slightly smaller than those in the compounds reported by Marder and co‐workers, which could be due to less steric hindrance in our compounds.^[47]^ It is noteworthy that the donor groups also exhibit a highly twisted configuration with respect to the bis(trifluoromethyl)phenyl group, with torsion angles of 75.3(2) and 76.6(2)° for **FMesB‐Cz**, 80.9(1)° for **FMesB‐Ac** and 83.2(1)° for **FMesB‐Ptz**. The above structural features reveal that two twisting nodes exist in these compounds, which can largely block the electron communication between the donor and acceptor, thus suppressing the charge‐transfer behaviour. In contrast, *p*‐NMe_2_‐^F^Xyl^F^Bf, with one twisting node between the trifluoromethylated borafluorene and the bis(trifluoromethyl)phenyl group, exhibits strong charge‐transfer character in its emission spectra.^[47]^ The shortest B⋅⋅⋅F distances are 2.518(9) and 2.575(9) Å for **FMesB‐Cz**, 2.512(4) and 2.611(4) Å for **FMesB‐Ac**, and 2.524(2) and 2.592(2) Å for **FMesB‐Ptz**, which are much shorter than the sum of the van der Waals radii of boron and fluorine (3.39 Å).^[54]^ This has previously been observed and discussed for boranes and boroles with *o*‐CF_3_‐aryl moieties[^34^, ^48^, ^50^, ^55^, ^56^] and can be explained by the lone‐pair electrons of the fluorine atoms interacting with the empty p orbital of the boron centre.

To gain a deeper understanding of these weak interactions, DFT calculations were conducted. We performed full geometry optimisations at the B3LYP/6‐31G** level of theory, starting from the crystal structure coordinates. In these optimised structures, the B⋅⋅⋅F distances of **FMesB‐Cz** and **FMesB‐Ac** are 2.620 and 2.618 Å, respectively. In the case of **FMesB‐Ptz**, the B⋅⋅⋅F distances are 2.615 Å for B1⋅⋅⋅F1 and 2.621 Å for B1⋅⋅⋅F4, because of the bending of the phenothiazine moiety. An atoms‐in‐molecules (AIM) analysis, performed with the Multiwfn software package,^[57]^ revealed that the electron densities (*ρ*) and its Laplacians (∇^2^ *ρ*) at the bond critical points (BCPs) of these molecules are *ρ*=0.0135 *ea*_0_^−3^ and ∇^2^ *ρ*=0.0514 *ea*_0_^−5^ (*a*_0_ is the Bohr radius) for **FMesB‐Cz**, *ρ*=0.0135 *ea*_0_^−3^ and ∇^2^ *ρ*=0.0506 *ea*_0_^−5^ for **FMesB‐Ac**, and *ρ*=0.0135 *ea*_0_^−3^ and ∇^2^ *ρ*=0.0506 *ea*_0_^−5^ (BCP1) and *ρ*=0.0134 *ea*_0_^−3^ and ∇^2^ *ρ*=0.0503 *ea*_0_^−5^ (BCP2) for **FMesB‐Ptz**. All of these values are small and comparable to those of ^F^Mes‐capped dithienylborane compounds, which indicates similar weak B⋅⋅⋅F interactions.^[50]^ In addition, typical π⋅⋅⋅π interactions with a head‐to‐tail packing mode are observed in the crystal structures of **FMesB‐Cz**, **FMesB‐Ac** and **FMesB‐Ptz**, with the distances between the borafluorene plane and the centroid of the respective benzene ring of the amine donor group being 3.524(6) and 3.805(6) Å as well as 3.583(7) and 3.815(7) Å for the two symmetrically independent molecules of **FMesB‐Cz**, respectively, and 3.570(3) and 3.732(3) Å for **FMesB‐Ac**. Due to the bending of the phenothiazine moiety, the molecular packing of **FMesB‐Ptz** shows a non‐parallel relationship between neighbouring molecules, which results in two packing distances between the phenothiazine and borafluorene moieties of around 3.482(1) and 4.091(1) Å, respectively (see Figure S1 in the Supporting Information).

### Photophysical and electrochemical properties

**Figure 4:**
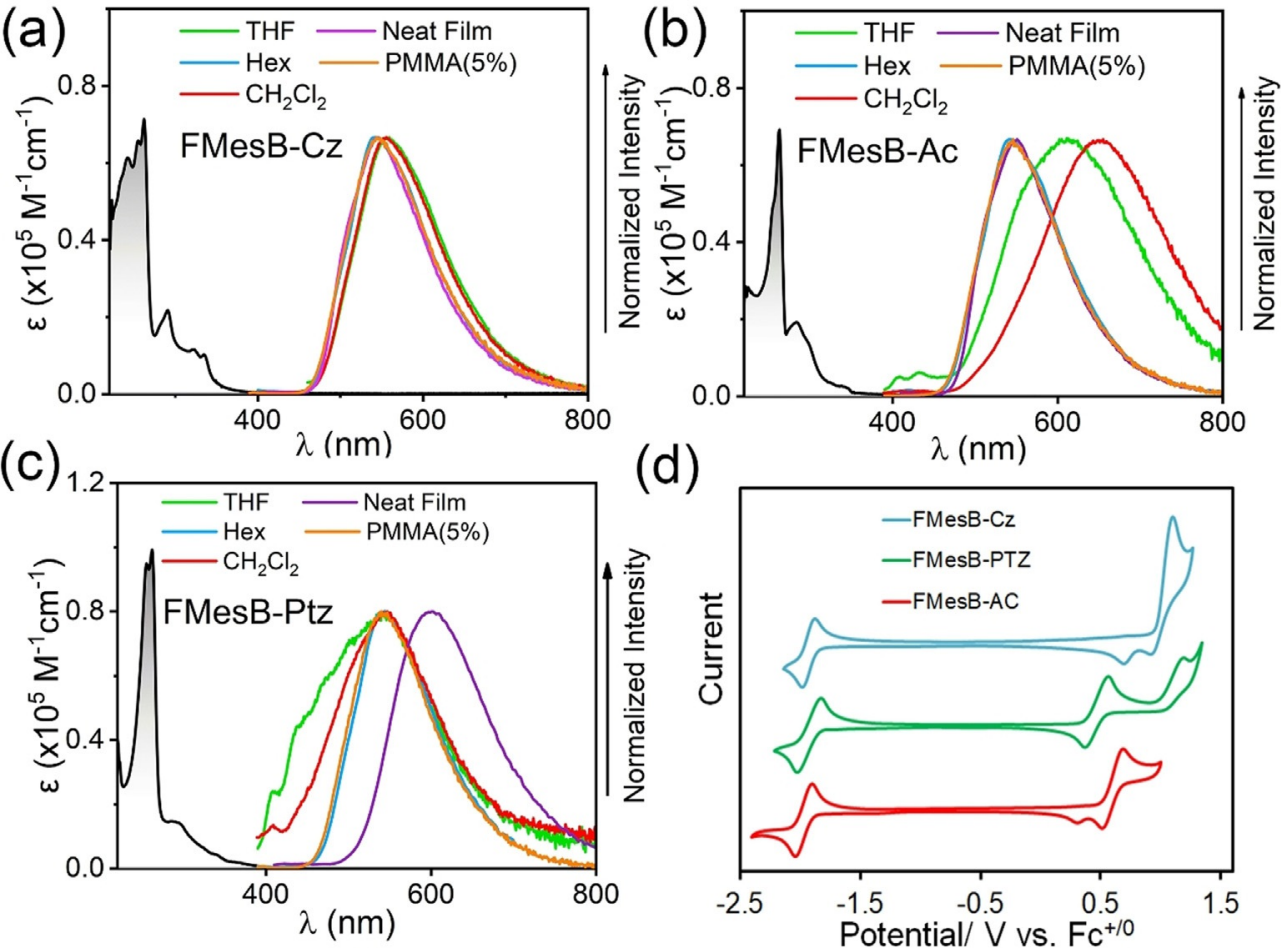
UV/Vis spectra (black line) of donor‐functionalised borafluorene compounds (a) **FMesB‐Cz**, (b) **FMesB‐Ac** and (c) **FMesB‐Ptz** in CH_2_Cl_2_, and photoluminescence spectra in different solvents (*c=*1×10^−5^ m) and in the solid state (colour lines, as indicated in the figures). (d) Cyclic voltammograms of the donor‐functionalised 9‐borafluorene compounds recorded in CH_2_Cl_2_ using *n*Bu_4_NPF_6_ (0.1 m) as the electrolyte and at a scan rate of 100 mV s^−1^.

The UV/Vis spectra of the compounds were recorded in CH_2_Cl_2_ solution (1×10^−5^ m); they exhibit an intense absorption at around 260 nm, and a weaker absorption at around 280–300 nm (Figure 4). All the compounds exhibit weak absorption in the visible range, with the onset value of the absorption tailing to beyond 400 nm.

The photoluminescent (PL) spectra show that these compounds exhibit bright‐yellow emission in hexane, with maxima in the range 545–550 nm, and relatively high quantum yields (PLQYs) (ca. 32 % for **FMesB‐Cz** and over 45 % for the other two compounds, Figure 4 and Table 1). Relatively long fluorescent lifetimes (134–140 ns in hexane) were observed for all of the compounds, which seems to be an intrinsic behaviour of borafluorenes and was reported by Rupar^[58]^ and Marder[^47^, ^59^] and their co‐workers. Almost no solvatochromism was observed in the emission spectra of **FMesB‐Cz**, but partial quenching was observed in more polar solvents (see Figure S2 in the Supporting Information). For compound **FMesB‐Ac**, solvatochromic behaviour was observed as the fluorescence maximum redshifted from 545 nm in hexane to around 650 nm in CH_2_Cl_2_ solution, with a significantly quenched intensity, similarly to that observed for the 9‐borafluorene compounds reported by Yamaguchi and co‐workers.^[44]^ In the case of **FMesB‐Ptz**, which has a stronger donating group, the fluorescence quantum yield dropped dramatically in polar solvents (e.g., ca. 1 % in CH_2_Cl_2_). Interestingly, all the compounds exhibit significantly quenched and blueshifted fluorescence in DMF solution, with the maximum emission wavelength shifted to around 400 nm for **FMesB‐Cz**, and around 450 nm for **FMesB‐Ac** and **FMesB‐Ptz** (see Figure S4). This phenomenon may be attributed to the coordination of the donor solvent DMF to the boron centre of the borafluorenes. A similar blueshift upon coordination to DMF, with increased luminescence, was observed by Yamaguchi et al.^[45]^ Other intramolecular^[56]^ and intermolecular^[47]^ adducts have been reported to exhibit excited‐state dissociation, retaining the emission wavelength of the “free” borafluorene, which indicates that the adducts of DMF with our compounds persist in their excited states. To confirm this hypothesis, we recorded the ^11^B NMR spectra of the borafluorene compounds in DMF (see Figure S4). In comparison with the ^11^B NMR chemical shifts in chloroform (ca. 65 ppm), the resonances of these compounds in DMF are shifted significantly upfield to around 11 ppm, revealing the formation of four‐coordinate boron centres.

**Table 1:** Photophysical properties of the donor‐functionalised borafluorenes.

Compd		298 K			78 K	
	*λ*_abs_ [nm]^[a]^	*λ*_PL_ [nm] sol^[b]^/film^[c]^	PLQY [%] sol^[b]^/film^[c]^	*τ* [ns] sol^[b]^/film^[c]^	*λ*_PL_ [nm]	*τ* [ns]
**FMesB‐Cz**	355	550/551	31.8/73.6	139.4/0.6, 107.9	534^[d]^/533^[e]^	3.7, 125.5^[d]^/60.8, 160.4^[e]^
**FMesB‐Ac**	355	545/551	45.4/57.3	140.3/0.6, 114.8	536^[d]^/534^[e]^	3.6, 121.8^[d]^/7.6, 155.7^[e]^
**FMesB‐Ptz**	390	548/606	46.3/22.0	134.1/0.3, 50.9	534^[d]^/525^[e]^	9.3, 124.0^[d]^/0.8, 167.6^[e]^

[a] Onset wavelength values of UV/Vis absorption of donor‐functionalised 9‐borafluorene compounds in hexane (1×10^−5^ m). [b] Data obtained in hexane. [c] Data obtained as neat films. [d] Data obtained at 78 K in a CH_2_Cl_2_ frozen glass (1×10^−5^ m). [e] Data obtained at 78 K in a 3‐methylpentane frozen glass (1×10^−5^ m).

The low‐temperature photophysical properties of the three compounds were also recorded in frozen 3‐methylpentane and CH_2_Cl_2_ at around 78 K (see Figure S6 in the Supporting Information and Table 1). The fluorescence maxima occur at 534–536 nm in CH_2_Cl_2_ and at 525–534 nm in 3‐methylpentane,and exhibit a well‐resolved band structure showing different vibrational modes. All of the compounds exhibit two lifetimes, as shown in Figure S6; one is shorter than the other typically by more than 100 ns, and the longer lifetimes are dominant. The long lifetimes of around 121–126 ns in frozen CH_2_Cl_2_ are slightly shorter than those in 3‐methylpentane (ca. 155–168 ns). The PL spectra of the compounds are almost identical, which indicates that the emissions can mainly be attributed to local excitation (LE) transitions localised on the 9‐borafluorene moiety.

In the solid state, both **FMesB‐Cz** and **FMesB‐Ac** exhibit bright‐yellow fluorescence in both the crystalline form and in neat films, with identical emission maxima at 551 nm, relatively high quantum yields of up to 73.6 % for **FMesB‐Cz** (Figure 4 and Table 1) and a lifetime of around 108 ns in neat films (see Figure S5 in the Supporting Information). In contrast, **FMesB‐Ptz** exhibits pale redshifted orange fluorescence both in the crystal and in the neat film, and the lifetime is shortened to 50.9 ns in the neat film. To interpret this phenomenon, 5 wt% doped poly(methyl methacrylate) (PMMA) films of all three compounds were prepared. The PL spectra of these film samples are almost identical to their PL spectra in hexane, and the lifetimes are also comparable with the data in hexane (see Figure S5), which indicates that the redshifted PL spectrum of **FMesB‐Ptz** in the neat film could be due to intermolecular interactions (e.g., the formation of excimers) in the excited state. The thermal stabilities of these compounds were determined by thermogravimetric analysis (TGA). The donor‐functionalised compounds exhibit much higher decomposition temperatures (>250 °C) than the compound without a donor (^**F**^ **MesBf**, *T*_d_=148 °C; see Figure S3). This excellent thermal stability indicates that the donor‐functionalised 9‐borafluorene compounds are promising candidates for applications in materials science.

The electrochemical properties of these 9‐borafluorenene derivatives were examined by cyclic voltammetry (CV) in CH_2_Cl_2_ (Figure 4 d). All of the compounds exhibit reversible reduction potentials, which can be assigned to the reduction of boron. The half‐wave reductive potentials (*E*_1/2_) of these compounds range from −1.90 to −1.95 V versus Fc^+/0^, which are slightly more negative than that of ^**F**^ **MesBF** (*E*_1/2_=−1.87 V vs. Fc^+/0^, see Figure S8 in the Supporting Information),^[49]^ which indicates that the electron‐donating groups weaken the electron‐accepting ability of boron, although the influence is very weak given the highly twisted geometries. In the case of oxidative processes, the phenothiazine‐terminated compound **FMesB‐Ptz** exhibits a reversible oxidation with *E*_1/2_=+0.46 V versus Fc^+/0^ and an irreversible oxidation with *E*_onset_=+1.01 V versus Fc^+/0^, whereas the other two compounds exhibit irreversible oxidations with *E*_onset_ values for **FMesB‐Ac** and **FMesB‐Cz** of +0.61 and +0.93 V versus Fc^+/0^, respectively. The HOMO and LUMO energies of these compounds were estimated from the oxidation and reduction potentials by using the equation *E*_LUMO/HOMO_=−5.16−*E*_red/ox_ (see Table 2).^[60]^

**Table 2:** Electrochemical data and calculated FMO energy levels for the donor‐functionalised borafluorenes.

Entry	*E*_red_ [V]	*E*_ox_ [V]	*E*_LUMO_ [eV]		*E*_HOMO_ [eV]	
			Exptl^[a]^	Calcd^[b]^	Exptl^[a]^	Calcd^[b]^
**FMesB‐Cz**	−1.93	0.93^[c]^	−3.23	−2.66	−6.09	−6.06
**FMesB‐Ptz**	−1.90	0.46	−3.26	−2.73	−5.62	−5.58
**FMesB‐Ac**	−1.95	0.61^[c]^	−3.21	−2.69	−5.77	−5.52

[a] *E*_LUMO/HOMO_=−5.16−*E*_red/ox_. [b] Calculated by DFT using Gaussian 09^[61]^ at the B3PW91/6‐311+G* level of theory. [c] Onset of the oxidation potential.

### Theoretical studies

**Figure 5:**
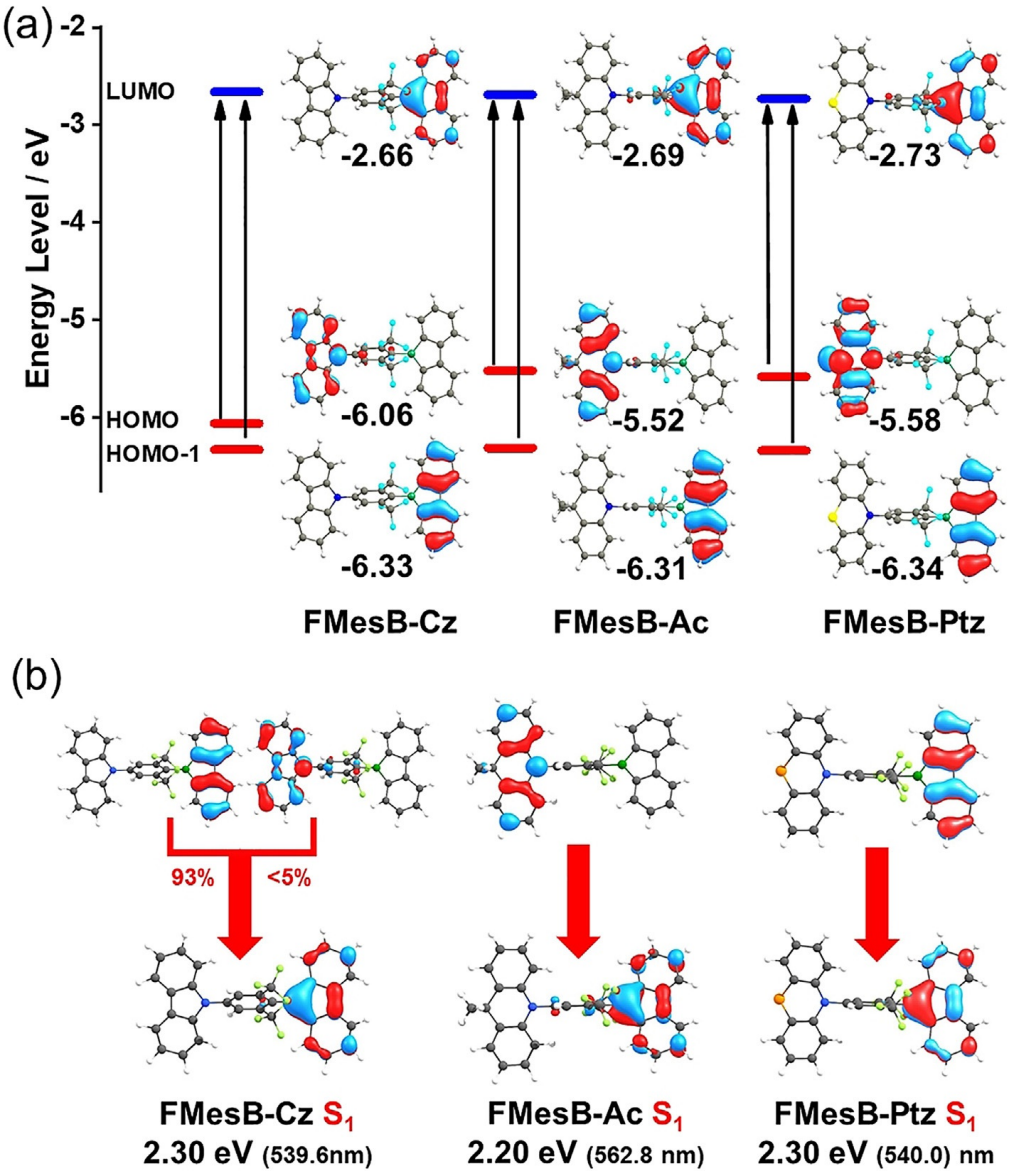
(a) Plots of the frontier orbitals and energy levels of the donor‐functionalised borafluorene derivatives (B3PW91/6‐311+G*//B3LYP/6‐31G**). (b) Optimised geometries and illustration of the transitions of the S1 states of the three compounds (PBE0/6‐31G**, using CH_2_Cl_2_ as solvent model).

DFT calculations were carried out on all of the compounds, with geometry optimisations conducted at the B3LYP/6‐31G** level of theory. Frequency calculations showed no imaginary frequencies, which indicates that energy minima had been reached. The energies of the frontier orbitals were generated by single‐point energy calculations based on the optimised structures, conducted at the B3PW91/6‐311+G* level of theory. As expected, the LUMOs of the compounds are located on the borafluorene moieties (Figure 5), and the HOMOs of the compounds in this work are mainly located on the donor groups without any contribution from the bis(trifluoromethyl)phenyl group, which could be due to the twisted configuration. In contrast, the HOMO of *p*‐NMe_2_‐^F^Xyl^F^Bf is distributed over both the dimethylamino and bis(trifluoromethyl)phenyl groups, because the coplanarity of the two groups is favourable for p–π conjugation.^[47]^ Generally, there is good agreement between the calculated and experimental HOMO energies. Although the experimentally approximated LUMO energies are almost identical for the three compounds, varying from −3.21 to −3.26 eV, the calculated energies show larger differences, ranging from −2.66 to −2.73 eV (Table 2). TD‐DFT calculations were conducted on all of the compounds at the PBE0/6‐311+G** level of theory (see Figure S11 and Table S6 in the Supporting Information).

For **FMesB‐Cz**, the weakly allowed S_0_→S_1_ (oscillator strength *f*=0.001) and S_0_→S_2_ (*f* = 0.03) transitions can be assigned to the HOMO−1→LUMO and HOMO→LUMO transitions, respectively. For **FMesB‐Ac** and **FMesB‐Ptz**, the S_0_→S_1_ and S_0_→S_2_ transitions can be assigned to HOMO→LUMO and HOMO−1→LUMO, respectively. The weak transition from the donor groups to the acceptor groups in these compounds could be due to the twisted spacer, bis(trifluoromethyl)phenyl, which reduces the overlap between the orbitals and thus reduces the oscillator strength of the transition. Optimisations of the S_1_ states were conducted for the three compounds at the PBE0/6‐31G** level of theory with additional solvent correction (CH_2_Cl_2_). As shown in Figure 5, the S_1_ state of **FMesB‐Ac** exhibits charge‐transfer character (mainly arising from a HOMO←LUMO transition), whereas **FMesB‐Cz** and **FMesB‐Ptz** reveal localised character with the transition to the S_1_ state being located on the borafluorene moiety (mainly HOMO−1←LUMO). These results match well the solvatochromic photoluminescent behaviour mentioned above.

Another important factor that can be obtained from DFT calculations is the antiaromaticity index of the 9‐borafluorene compounds. We calculated the nucleus independent chemical shifts (NICSs) as an index of aromaticity by means of DFT at the B3LYP/6‐31G** level of theory based on the optimised structures.[^62^, ^63^] NICS(1)_*zz*_ values, which represent the NICS value 1 Å above and below the centre of the ring, are listed in Figure S10 in the Supporting Information. The results indicate that the NICS(1)_*zz*_ values of the borole rings are around +26, which is almost identical to that of ^**F**^ **MesBf** (NICS(1)_*zz*_=+26.05, calculated by using the same method as us), and indicates moderate antiaromaticity. This value is similar to the NICS(1)_*zz*_ values previously reported for other borafluorene derivatives.^[35]^ These results suggest that the electron‐donating groups have almost no influence on the aromaticity of the borole ring.

### Fabrication of OLEDs based on the donor‐functionalised borafluorene compounds

**Figure 6:**
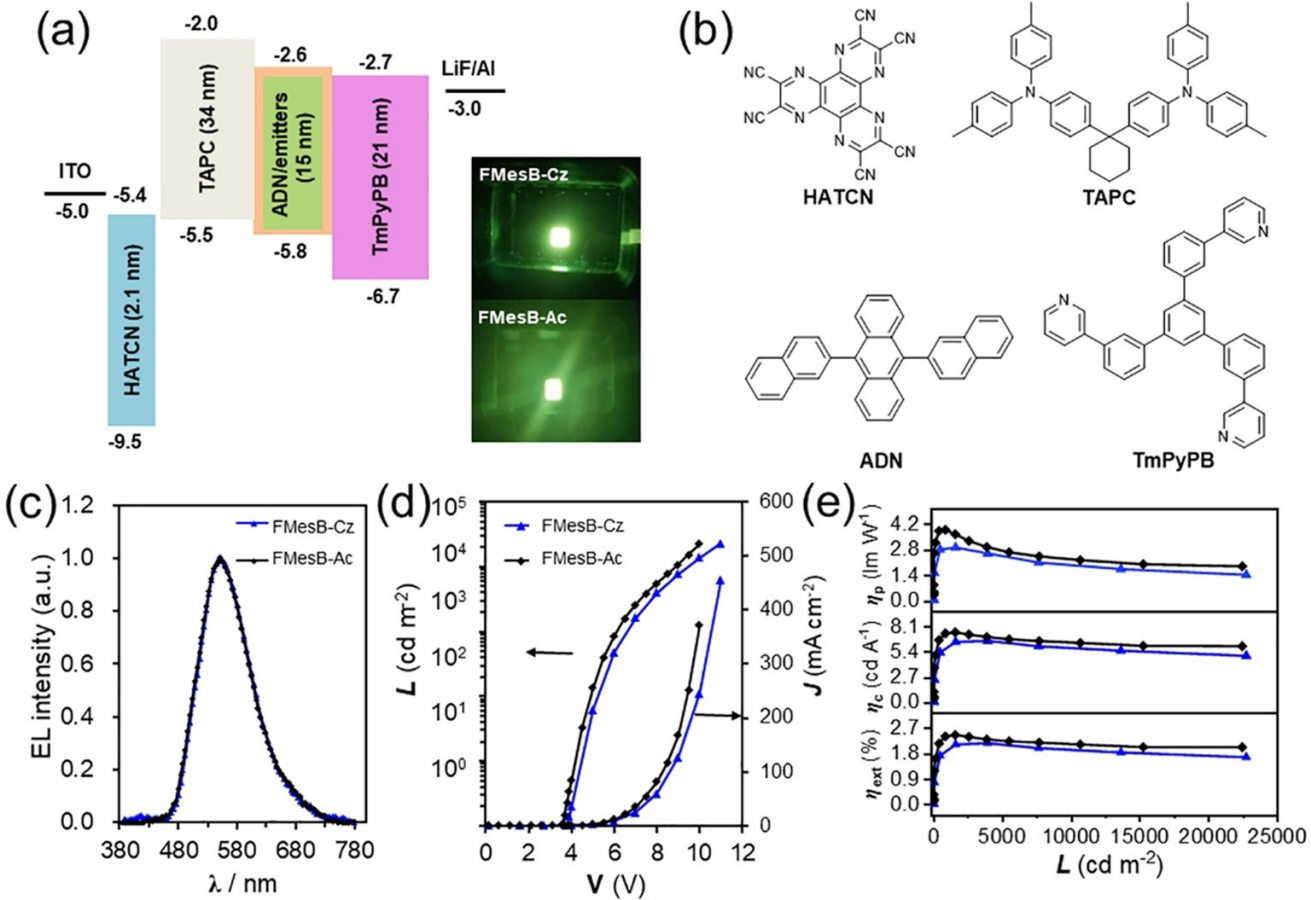
EL characteristics of OLED devices based on **FMesB‐Cz** and **FMesB‐Ac**. (a) Configuration and energy diagram of the devices, and photographs showing their emission colours. (b) Structures of the molecules used in the devices. (c) EL spectra of the devices at a luminance of around 1000 cd m^−2^. (d) Luminance (*L*)–voltage (*V*)–current density (*J*) characteristics for the two devices. (e) Power efficiency (*η*_p_), current efficiency (*η*_c_) and external quantum efficiency (*η*_ext_) versus luminance (*L*) curves for the two devices.

To explore the application of these functionalised borafluorenes in organic electronics, vacuum‐deposited OLEDs based on **FMesB‐Cz** and **FMesB‐Ac** as emitting dopants were fabricated to investigate their electroluminescence (EL) performance. In this work, 4,4′‐cyclohexylidenebis[*N*,*N*‐bis(4‐methylphenyl)aniline] (TAPC) was used as the hole transport material, 1,4,5,8,9,11‐hexaazatriphenylenehexacarbonitrile (HATCN) was used as the hole injection layer material, 1,3,5‐tris[3‐(3‐pyridyl)phenyl]benzene (TmPyPB) was used as the electron‐transport material and 9,10‐di(2‐naphthyl)anthracene (ADN) was used as the host material. Devices with the configuration ITO/HATCN (2.1 nm)/TAPC (34 nm)/5 wt% emitting dopant:ADN (15 nm)/TmPyPB (21 nm)/LiF (1 nm)/Al (100 nm) were fabricated and characterised. As shown in Figure 6 and Table S2 in the Supporting Information, the devices based on **FMesB‐Cz** and **FMesB‐Ac** achieved turn‐on voltages of between 3.6 and 3.8 V, and produced EL spectra exhibiting a similar yellow‐green colour with identical emission peaks at 552 nm, resembling their PL spectra in the solid state. The devices based on **FMesB‐Cz** and **FMesB‐Ac** achieved a maximum luminance of 22 710 cd m^−2^ at 11 V and 22 410 cd m^−2^ at 10 V, respectively. The maximum external quantum efficiency (2.4 %) and current efficiency (7.5 cd A^−1^) of the **FMesB‐Ac**‐based device were higher than those of the **FMesB‐Cz**‐based device. These results suggest that the better EL properties of **FMesB‐Ac** may be attributed to its higher solid‐state efficiency. Furthermore, both devices exhibited excellent performances, with external quantum efficiencies of up to 2.1 % at 1000 cd m^−2^, which are comparable to those reported for other boron‐containing OLED devices.[^64^, ^65^, ^66^, ^67^, ^68^]

## Conclusion

A series of 9‐borafluorene derivatives functionalised with carbazole, acridine and phenothiazine as donor groups with a bis(trifluoromethyl)phenyl spacer have been prepared in moderate yields, and all of the compounds exhibited good stability. The crystal structures of these compounds indicate that the bis(trifluoromethyl)phenyl group inhibits the donor–acceptor interaction to some extent. Furthermore, typical π‐stacking interactions are observed between the borafluorene and donor groups of neighbouring molecules in the solid state. Electrochemical studies and DFT calculations indicate that the introduction of donor groups influences the energy levels of the borafluorene group only slightly. Although the compounds are almost colourless in dilute solution, which is attributed to the weakly allowed S_0_→S_1_ transition, some of the compounds exhibit strong yellowish emission in solution and the solid state with quantum yields of up to 73.6 % for **FMesB‐Cz** in neat film. **FMesB‐Ptz** shows a relatively weak and redshifted emission in the neat film and crystal, which could be attributed to intermolecular interactions. All of the compounds exhibit excellent thermal stability with much higher decomposition temperatures than the non‐donor analogue, which demonstrates their potential for application in materials. OLED devices were fabricated with two of the highly emissive compounds as light‐emitting materials. Both devices exhibited strong electroluminescence at 550 nm, and a maximum luminance intensity greater than 22 000 cd m^−2^. This is the first example of borafluorene derivatives being used as light‐emitting materials in OLED devices. Finally, this study has demonstrated that the strategy of balancing the stability and intrinsic properties of 9‐borafluorene greatly expands its prospects of application in the field of materials.

## Experimental Section

**Crystallographic data**: Deposition numbers 2000574 (**FMesB‐Cz**), 2000564 (**FMesB‐Ac**), and 2000565 (**FMesB‐Ptz**) contain the supplementary crystallographic data for this paper. These data are provided free of charge by the joint Cambridge Crystallographic Data Centre and Fachinformationszentrum Karlsruhe Access Structures service.

## Conflict of interest

The authors declare no conflict of interest.

## Supporting information

As a service to our authors and readers, this journal provides supporting information supplied by the authors. Such materials are peer reviewed and may be re‐organized for online delivery, but are not copy‐edited or typeset. Technical support issues arising from supporting information (other than missing files) should be addressed to the authors.

Supplementary
