# Supplementary material for: Highly Emissive 9‐Borafluorene Derivatives: Synthesis, Photophysical Properties and Device Fabrication
Source: Chemistry. 2021 Mar 10;27(20):6274–82. doi: 10.1002/chem.202005185 (PMC8048904; doi:10.1002/chem.202005185)
Supplement: Supplementary file 1 — Supplementary [file CHEM-27-6274-s001.pdf]

Chemistry–A European Journal

Supporting Information

# Highly Emissive 9-Borafluorene Derivatives: Synthesis, Photophysical Properties and Device Fabrication

Xing Chen,[a] Guoyun Meng,[a] Guanming Liao,[a] Florian Rauch,[b] Jiang He,[b] Alexandra Friedrich,[b] Todd B. Marder,*[b] Nan Wang,[a] Pangkuan Chen,[a] Suning Wang,[a, c] and Xiaodong Yin*[a]

# Supporting Information

# Table of Contents

**S1. Materials and General Methods** ........................................................................................1

**S2. Synthetic Procedure** ........................................................................................2

**S3. Crystal Structures** ........................................................................................5

**S4. Photophysical and Electrochemical Data** ........................................................................................7

**S5. DFT Calculations** ........................................................................................11

**S6. NMR Spectra and HRMS Data** ........................................................................................18

**S7. References** ........................................................................................23

## S1. Materials and General Methods.

The compounds 2,2'-dibromobiphenyl, n-butyllithium, boron trichloride (1.0 mol/L in toluene), carbazole, phenothiazine, 9,9-dimethyl-9,10-dihydroacridine, 1-iodo-3,5-bis(trifluoromethyl)benzene, 1-bromo-3,5-bis(trifluoromethyl)benzene, dichloromethylstannane, were purchased from Energy Chemical. Tetrahydrofuran, petroleum ether, dichloromethane, methanol, and ethyl acetate were purchased from Sinopharm Chemical Reagent Co., Ltd. These chemicals were used without further purification unless otherwise noted. Anhydrous solvents were distilled from commercial solvent using sodium/benzophenone. All commercial chemicals were also used without further purification.

NMR spectra were obtained on a Bruker Ascend 400M spectrometer (Avance III HD 400 MHz) operating at 400 MHz for $^{1}H$ NMR, 101 MHz for $^{13}C$ NMR, and 128 MHz for $^{11}B$ NMR in $CDCl_3$ unless otherwise noted. $^{1}H$ NMR chemical shifts were referenced to residual $CHCl_3$ (7.26 ppm), $^{13}C$ NMR (proton decoupled) chemical shifts were referenced to $CDCl_3$ (77.16 ppm). For $^{11}B$ NMR spectra, boron-free quartz NMR tubes were used and the spectra were referenced to external $BF_3 \cdot Et_2O$ ($\delta = 0$). High resolution mass spectral data were obtained on an Agilent (Q-TOF 6520) mass spectrometer. UV-Vis absorption spectra were recorded on a Cary 300 UV-Vis spectrophotometer. Luminescent spectra were obtained on an Edinburgh Instruments FLS980 or Lengguang Tech F97PrO spectrophotometer. Fluorescent quantum efficiencies were determined with a Hamamatsu C11347-11 Quantaurus-QY spectrometer.

DFT calculations were performed with the Gaussian 09 D.01 program package.[1] Geometry optimizations were conducted at the B3LYP/6-31G** level of theory, and single point energies were calculated at the B3PW91/6-311+G* level of theory. Vertical transitions were calculated using TD-DFT (pbe0/6-311+G**).

**Device Fabrication and Measurement:** All OLEDs were fabricated on glass substrates coated with a patterned transparent ITO (indium tin oxide) conductive layer. The ITO coated substrates were cleaned by successively sonicating in a detergent solution, distilled water, acetone, and isopropanol in an ultrasonic bath. Prior to use, each ITO glass was cleaned by rinsing with acetone and isopropanol. Subsequently, the surface of the ITO substrate was dried for 5 min in an oven at 393 K. After 10 min of ozone plasma treatment, using a Harrick Plasma PDC-32G-2, 100W Plasma Cleaner, the substrates were loaded into a deposition chamber with a vacuum < $5.0 \times 10^{-4}$ Pa during the evaporation process. All organic materials were thermally evaporated at a deposition rate of 0.2 − 0.3 Å $s^{-1}$ using a JD400C multisource organic molecular vapour deposition system from Jiuda Vacuum Technology Co., Ltd. A LiF layer was deposited at a rate of 0.05 Å $s^{-1}$ and the Al cathode at a rate of 3 − 5 Å $s^{-1}$. The thickness of each layer was characterised in advance on a Bruker Dektak XT surface profiler. The active area of the diode segments was $2 \times 2$ $mm^2$. The OLED characteristics of all fabricated devices were evaluated at room temperature under a nitrogen atmosphere inside a glovebox using a Photo Research PR-655spectrophotometer, with a Keithley model 2400 computer controlled programmable direct-current power supply voltage-current source.

## S2. Synthetic Procedure

### Part 1. Synthesis of Donor-Functionalized Fluorinated Aryl Compounds

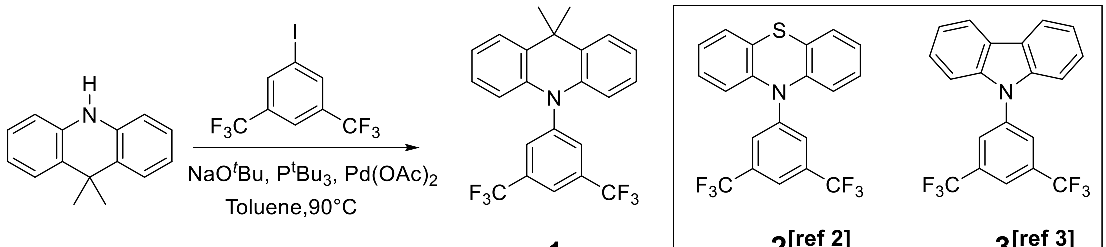


1 2[ref 2] 3[ref 3]

**Part 2 Synthesis of Donor-Functionalized Borafluorene Compounds**

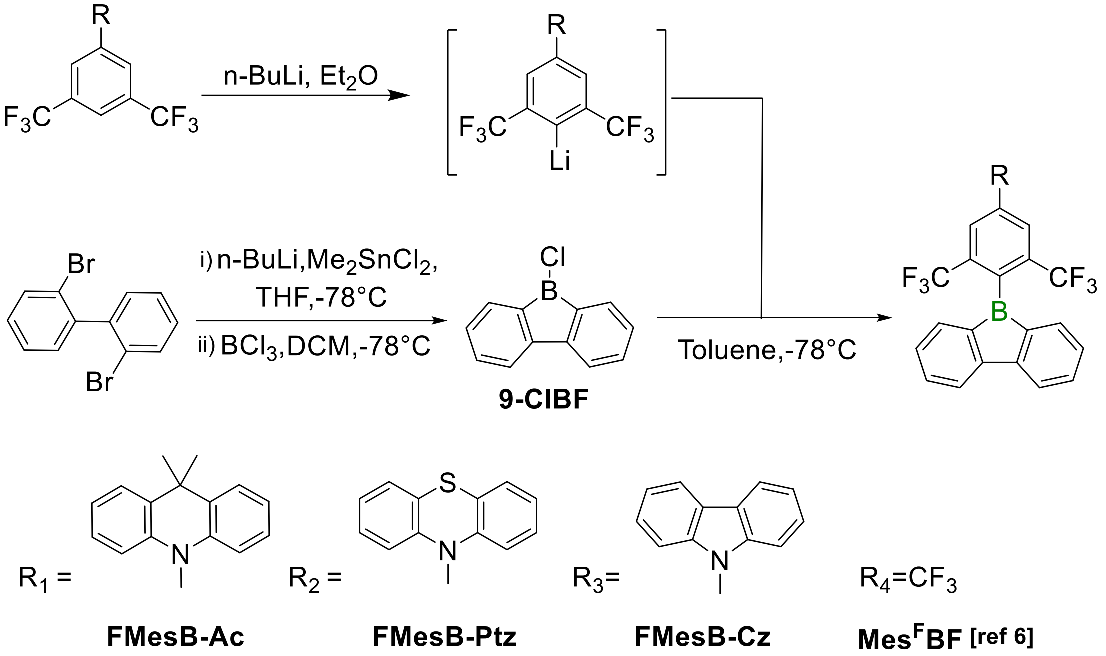


## 2.1 Synthesis of Donor-Functionalised Fluorinated Aryl Compounds

**2.1.1 Synthesis of 10-(3,5-bis(trifluoromethyl)phenyl)-9,9-dimethyl-9,10-dihydroacridine (1)** 9,9-Dimethylacridan (0.21 g, 1.0 mmol), 1-iodo-3,5-bis(trifluoromethyl)benzene (0.64 g, 2.0 mmol), sodium tert-butoxide (0.096 g, 1.0 mmol), tris(1,1- dimethylethyl)phosphine (1.0 M solution in toluene, 0.2 mL) and palladium acetate (20 mg, 0.09 mmol) were suspended in 20 mL of toluene, and stirred under an Ar flow in a 100 mL three-neck flask for 20 min. Then, the mixture was heated at 90 ℃ with stirring for 12 h. The mixture was extracted with dichloromethane and then washed with deionized water. The organic phase was concentrated and purified by column chromatography to obtain the product 10-(3,5-bis(trifluoromethyl)phenyl)-9,9-dimethyl-9,10-dihydroacridine (**1**) (0.235g, 56%) as a white powder. $^{1}$H NMR (400 MHz, $CDCl_3$) δ 8.04 (s, 1H), 7.87 (s, 2H), 7.53–7.48 (m, 2H), 7.06–6.98 (m, 4H), 6.19–6.09 (m, 2H), 1.71 (s, 6H); $^{13}$C{$^{1}$H} NMR (101 MHz, $CDCl_3$) δ 143.3 140.2, 134.8 (q, $J_{C\text{-}F}$ = 34 Hz), 132.3, 132.3, 130.9, 126.8, 125.8, 123.0 (q, $J_{C\text{-}F}$ = 273 Hz), 122.2, 121.8, 114.0, 36.2, 31.3; ESI-HRMS (m/z): calcd. For $C_{23}H_{17}F_6N$ $[M+H]^+$ 422.1343, found 422.1348.

The compounds 10-(3,5-bis(trifluoromethyl)phenyl)-10H-phenothiazine (**2**)[2], and 9-(3,5-bis(trifluoromethyl)phenyl)-9H-carbazole (**3**)[3] were synthesised according to literature methods.

## 2.2 Synthesis of Donor-Functionalized Borafluorene Compounds

**2.2.1 Synthesis of 9-chloro-9-borafluorene**

The compound 9-chloro-9-borafluorene (**9-ClBF**) was synthesised according to the literature.[4]

A Schlenk tube (50 ml) was charged with 9,9-dimethyl-9-stannafluorene[5] (1.0g, 3.3 mmol) and $CH_2Cl_2$ (10 mL). A 1.0 M toluene solution of $BCl_3$ (3.3 mL, 3.3 mmol) was added dropwise to the solution at

–78 ℃. The mixture was allowed to warm to room temperature overnight. All volatiles were removed under vacuum to yield a yellow powder. The crude product of 9-chloro-9-borafluorene (**9-ClBF**) was used directly in the next step without purification.

**2.2.2 Synthesis of FMesB-Ac**, **FMesB-Ptz**, **FMesB-Cz.**

**General synthetic procedure for lithiated compounds 1Li, 2Li, 3Li**: A solution of 10-(3,5-bis(trifluoromethyl)phenyl)-9,9-dimethyl-9,10-dihydroacridine (**1**) (2.05 g, 4.95 mmol) in diethyl ether (30 mL) was treated with *n*-BuLi (1.6 M, 5.94 mmol) at −78 °C. The reaction was allowed to stir at −78 ℃ for 30 min and then gradually warmed to room temperature. After 3 h at room temperature, the diethyl ether was removed under vacuum to yield a red powder. The materials were used for further reactions without further purification.

**General procedure for the synthesis of compounds FMesB-Ac**, **FMesB-Ptz**, **and FMesB-Cz,**: The lithiated materials **1Li, 2Li, 3Li,** were suspended in toluene and cooled to −78 °C. A solution of 9-chloro-9-borafluorene (670 mg, 3.3 mmol) in toluene (10 mL) was added dropwise and the reaction was allowed to warm to room temperature gradually overnight. The mixture was extracted with $CH_2Cl_2$ and washed with deionized water. The organic phase was separated and purified by column chromatography to obtain **10-(4-(5H-dibenzo[b,d]borol-5-yl)-3,5-bis(trifluoromethyl)phenyl)-9,9-dimethyl-9,10-dihydroacridine** (**FMesB-Ac**) (410 mg, 21%) as a yellow powder. $^{1}$H NMR (400 MHz, $CDCl_3$) δ 7.94 (s, 2H), 7.53 (d, *J* = 8 Hz, 2H), 7.45–7.35 (m, 4H), 7.31 (d, *J* = 7 Hz, 2H), 7.14–7.07 (m, 4H), 7.03 (t, *J* = 7 Hz, 2H), 6.26 (d, *J* = 8 Hz, 2H), 1.73 (s, 6H). $^{13}$C{$^{1}$H} NMR (101 MHz, $CDCl_3$) δ 153.6, 142.6, 142.0(br), 140.4, 135.6 (q, $J_{C\text{-}F}$ = 32 Hz), 135.0, 134.4, 132.1(br), 131.1, 128.7, 126.9, 125.8, 123.9 (q, $J_{C\text{-}F}$ = 275 Hz), 121.9, 120.0, 114.1, 36.3, 31.3. $^{11}$B NMR (128 MHz, $CDCl_3$) δ 65.5. ESI-HRMS (m/z): calcd. For $C_{35}H_{24}BF_6N$ $[M+H]^+$ 584.1984, found 584.1990.

**10-(4-(5H-dibenzo[b,d]borol-5-yl)-3,5-bis(trifluoromethyl)phenyl)-10H-phenothiazine (FMesB-Ptz)**, was obtained as an orange-yellow powder. $^{1}$H NMR (400 MHz, $CDCl_3$) δ 7.65 (s, 2H), 7.41 (d, *J* = 1.2 Hz, 2H), 7.40–7.38 (m, 2H), 7.37 (s, 2H), 7.35–7.26 (m, 4H), 7.22 (d, *J* = 7 Hz, 2H), 7.17 (t, *J* = 7 Hz, 2H), 7.10 (d, *J* = 9 Hz, 2H), 7.05 (t, *J* = 7 Hz, 2H). $^{13}$C{$^{1}$H} NMR (101 MHz, $CDCl_3$) δ 153.4, 145.5, 142.3(br), 141.6, 134.7, 134.3 (q, *J* = 32 Hz), 134.3, 130.7, 128.8, 128.5, 127.7, 125.9, 124.2 (q, $J_{C\text{-}F}$ = 273 Hz), 123.7, 120.1(br), 119.8. $^{11}$B NMR (128 MHz, $CDCl_3$) δ 67.6. ESI-HRMS (m/z): calcd. For $C_{32}H_{18}BF_6NS$ $[M+H]^+$ 574.1248, found 574.1235.

**9-(4-(5H-dibenzo[b,d]borol-5-yl)-3,5-bis(trifluoromethyl)phenyl)-9H-carbazole (FMesB-Cz)**, was obtained as a yellow powder. $^{1}$H NMR (400 MHz, $CDCl_3$) δ 8.19 (t, *J* = 4 Hz, 3H), 7.55–7.47 (m, 4H), 7.4 –7.35 (m, 6H), 7.31 (d, *J* = 7 Hz, 2H), 7.11 (t, *J* = 7 Hz, 2H). $^{13}$C{$^{1}$H} NMR (101 MHz, $CDCl_3$) δ 153.6, 142.1 (br), 140.2, 139.1, 135.0, 134.7 (q, *J* = 32 Hz), 134.4, 128.7, 127.0, 126.7, 124.2, 123.9 (q, $J_{C\text{-}F}$ = 273 Hz), 121.3, 120.9, 120.0, 109.4. $^{11}$B NMR (128 MHz, $CDCl_3$) δ 66.9. ESI-HRMS (m/z): calcd. For $C_{32}H_{18}BF_6N$ $[M]^+$ 541.1436, found 541.1484.

The compound **Mes$^{F}$BF** was synthesised for reference according to the literature method.[6]

## S3. Crystal Structures

Single-crystal X-ray diffraction data were collected on a Bruker D8 Venture 4-circle diffractometer using Mo-K$\alpha$ radiation ($\lambda$ = 0.71073 Å) at 180 K for **FMesB-Ac** and **FMesB-Ptz**, and at 298 K for **FMesB-Cz**. The images were processed and corrected for Lorentz-polarisation effects and absorption as implemented in the Bruker software packages. The structures were solved using the intrinsic phasing method (SHELXT)[7] and Fourier expansion technique. All non-hydrogen atoms were refined in anisotropic approximation, with hydrogen atoms 'riding' in idealised positions, by full-matrix least squares against $F^2$ of all data, using SHELXL software.[8] Hydrogen atoms were refined with isotropic displacement parameters. Olex2[9] was used as a graphical user interface and for the preparation of the CIF files. The crystal of **FMesB-Cz** was a non-merohedral twin with twin domains rotated by 179.8 ° around real axis [1 0 0]. Crystal data and experimental details are listed in Table S1; full structural information has been deposited with the Cambridge Crystallographic Data Centre: CCDC numbers are 2000574 **(FMesB-Cz**), 2000564 (**FMesB-Ac**), and 2000565 (**FMesB-Ptz**).

**Table S1.** Crystal data and structure refinement for **FMesB-Ptz**, **FMesB-Ac**, and **FMesB-Cz**.

| Identification code | **FMesB-Ptz** | **FMesB-Ac** | **FMesB-Cz** |
|---|---|---|---|
| CCDC | 2000565 | 2000564 | 2000574 |
| Empirical formula | $C_{32}H_{18}BF_6NS$ | $C_{35}H_{24}BF_6N$ | $C_{32}H_{18}BF_6N$ |
| Formula weight / g·mol$^{-1}$ | 573.34 | 583.36 | 541.28 |
| Temperature / K | 180(2) | 180(2) | 296(2) |
| Radiation, λ / Å | Mo-Kα, 0.71073 | Mo-Kα, 0.71073 | Mo-Kα, 0.71073 |
| Crystal system | orthorhombic | orthorhombic | monoclinic |
| Space group | *Pbcn* (no. 60) | *Pbca* (no. 61) | *Pc* (no. 7) |
| *a* / Å | 30.4800(17) | 12.9134(11) | 15.654(3) |
| *b* / Å | 13.1979(7) | 12.6389(13) | 13.321(2) |
| *c* / Å | 12.5614(7) | 33.369(3) | 12.157(4) |
| $\alpha$ / ° | 90 | 90 | 90 |
| $\beta$ / ° | 90 | 90 | 98.668(11) |
| $\gamma$ / ° | 90 | 90 | 90 |
| Volume / Å$^3$ | 5053.1(5) | 5446.1(9) | 2506.1(10) |
| *Z* | 8 | 8 | 4 |
| $\rho_{calc}$ / g·cm$^{-3}$ | 1.507 | 1.423 | 1.435 |
| $\mu$ / mm$^{-1}$ | 0.197 | 0.111 | 0.114 |
| *F*(000) | 2336 | 2400 | 1104 |
| Crystal size / mm$^3$ | 0.48 × 0.37 × 0.10 | 0.26 × 0.20 × 0.03 | 0.25 × 0.15 × 0.08 |
| Crystal color, habit | yellow block | yellow block | yellow block |
| 2Θ range / ° | 4.672 - 55.118 | 4.672 - 50.244 | 3.058 - 50.246 |
| Index ranges | -39 ≤ h ≤ 39, -14 ≤ k ≤ 17, -16 ≤ l ≤ 16 | -15 ≤ h ≤ 14, -15 ≤ k ≤ 14, -39 ≤ l ≤ 39 | -18 ≤ h ≤ 18, -15 ≤ k ≤ 15, -14 ≤ l ≤ 14 |
| Reflections collected | 67021 | 59178 | 25663 |
| Independent reflections | 5818 | 4854 | 6616 |
| $R_{int}$ | 0.0853 | 0.1666 | 0.0371 |
| $R_\sigma$ | 0.0421 | 0.0721 | 0.0312 |
| Parameters/restraints | 370/0 | 390/0 | 722/2 |
| GooF on $F^2$ | 1.028 | 1.051 | 1.112 |
| $R_1$ [I>=2σ (I)] | 0.0423 | 0.0612 | 0.0458 |
| $wR^2$ [all data] | 0.1169 | 0.1652 | 0.1126 |
| Max. / min. residual electron density / e Å$^{-3}$ | 0.29/-0.32 | 0.34/-0.26 | 0.22/-0.17 |
| Flack Parameter | N/A | N/A | 0.5(7) |

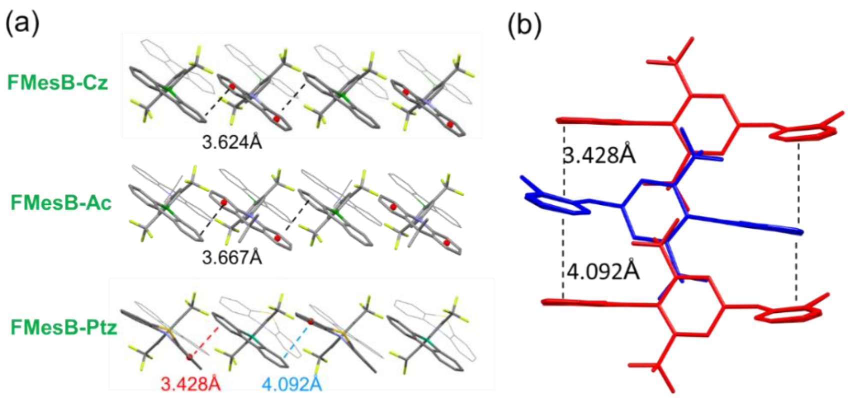


**Figure S1.** (a) Crystal packing mode of **FMesB-Cz**, **FMesB-Ac**, and **FMesB-Ptz**; (b) side view of **FMesB-Ptz** packing mode.

## S4. Photophysical and Electrochemical Data

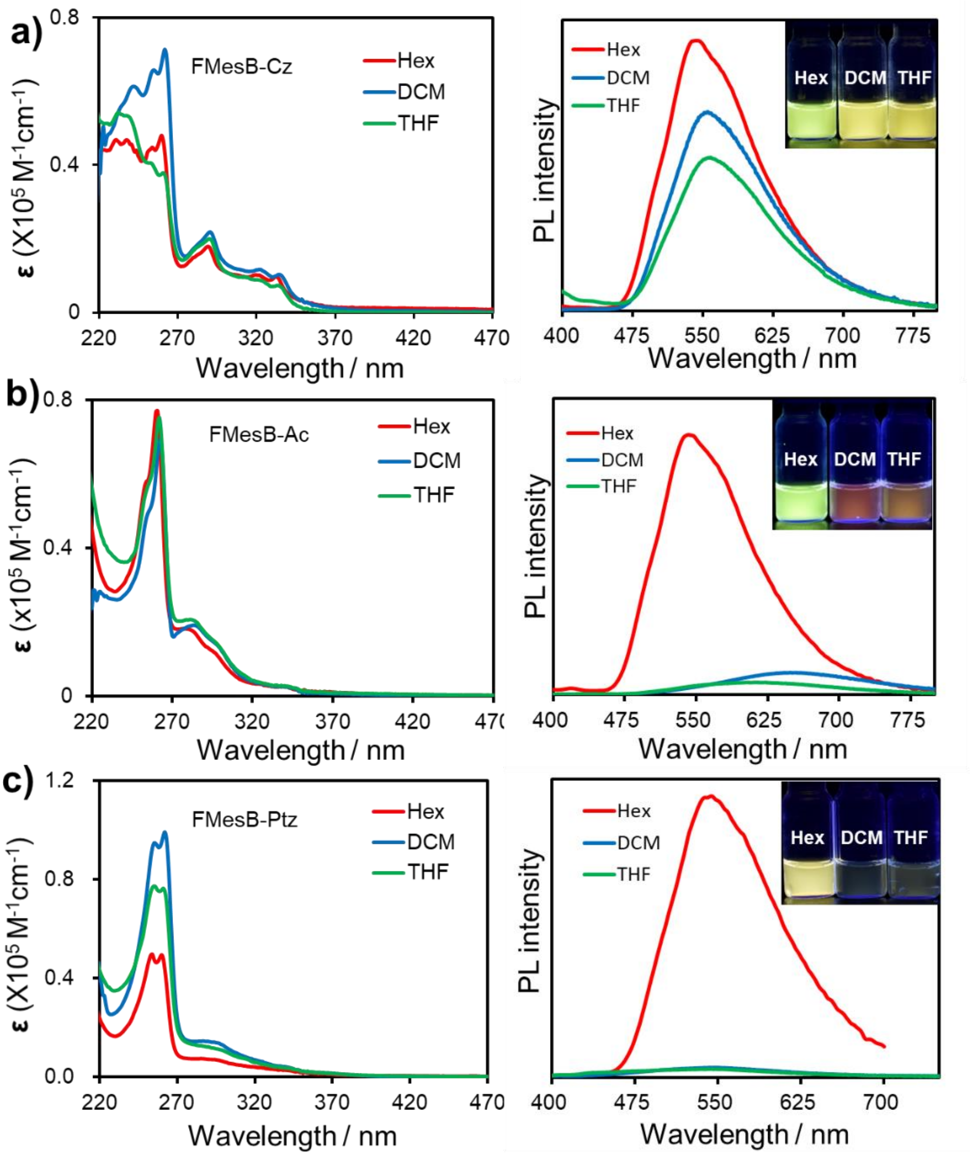


**Figure S2**. UV-Vis absorption spectra and fluorescence spectra of **FMesB-Ac, FMesB-Ptz,** and **FMesB-Cz** in different solvents (Hexane, $CH_2Cl_2$, and THF; c=0.01 mM, air). Inset: photo of these compounds in different solvents under a UV lamp (365 nm).

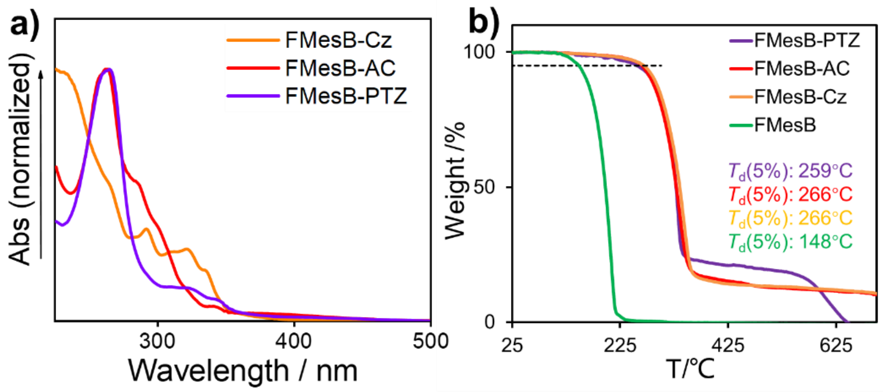


**Figure S3**. (a) Normalized UV-Vis absorption spectra of **FMesB-Cz**, **FMesB-Ac**, and **FMesB-Ptz**; (b) Thermogravimetric diagrams of of **FMesB-Cz**, **FMesB-Ac**, **FMesB-Ptz**, and **Mes$^F$BF** ( $N_2$,10 °C/min ).

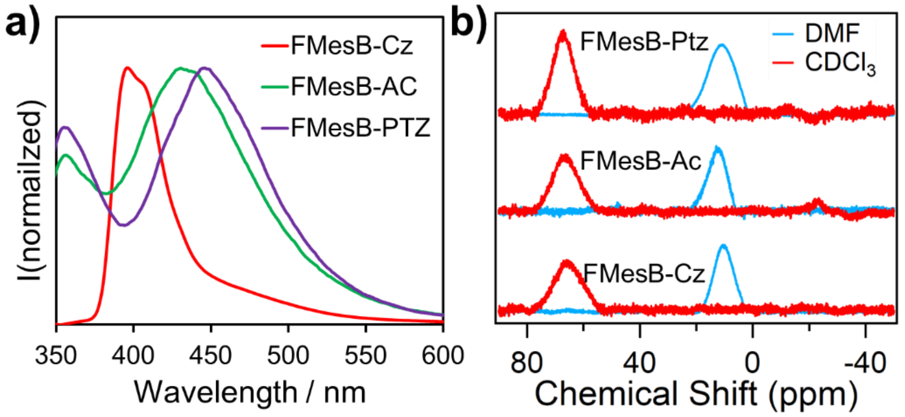


**Figure S4**. (a) Normalized fluorescence spectra of **FMesB-Cz, FMesB-Ac, FMesB-Ptz** in DMF; (b) $^{11}B$ NMR spectrum of **FMesB-Cz, FMesB-Ac, FMesB-Ptz** (in $CDCl_3$ and DMF, 225 MHz).

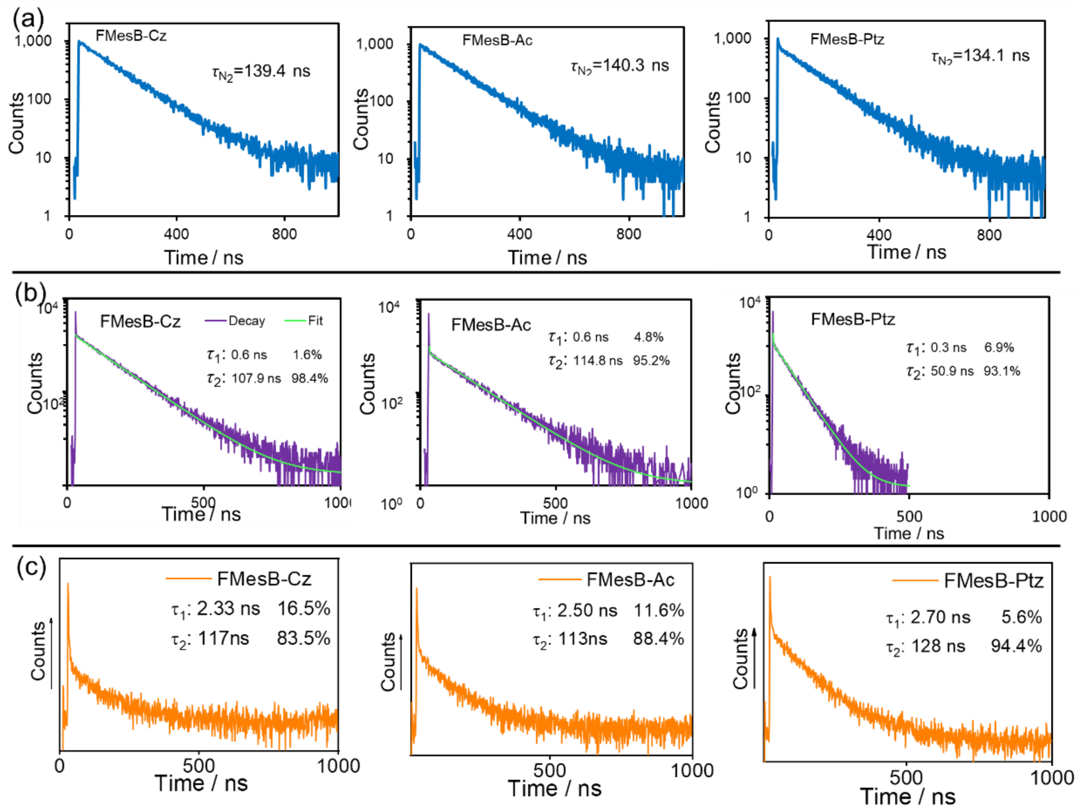


**Figure S5**. (a) Transient PL decay curves in oxygen-free hexane at 298 K; (b) Transient PL decay curves in neat film at 298 K; (c) Transient PL decay curves in 5% doped PMMA film at 298 K.

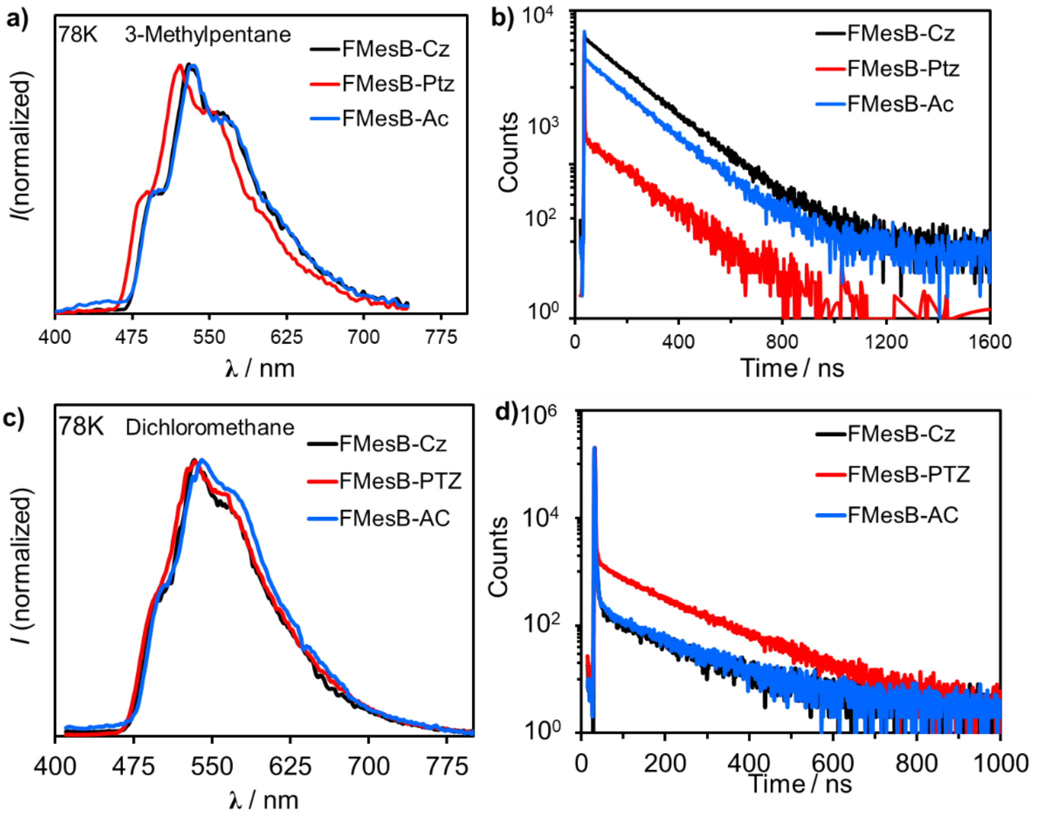


| | τ (ns) | | |
|---|---|---|---|
| | **FMesB-Cz** | **FMesB-Ac** | **FMesB-Ptz** |
| **$CH_2Cl_2$** | 3.7(23.2%),125.5 (76.8%) | 3.6 (20.2%),121.8 (79.8%) | 9.3(6.4%),124.0 (93.6%) |
| **3-Methylpentane** | 60.8 (1.4%),160.4 (98.6%) | 7.6(0.6%), 155.7 (99.4%) | 0.8(8.5%),167.6 (91.5%) |

**Figure S6**. (a) Photoluminescence spectra in 3-methylpentane at 78 K; (b)Transient PL decays of donor-functionalised borafluorene compounds in 3-methylpentane at 78 K; (c) Photoluminescence spectra in $CH_2Cl_2$ at 78 K; (d) Transient PL decays of donor-functionalised borafluorene compounds in $CH_2Cl_2$ at 78 K. Fitting data is listed in the table.

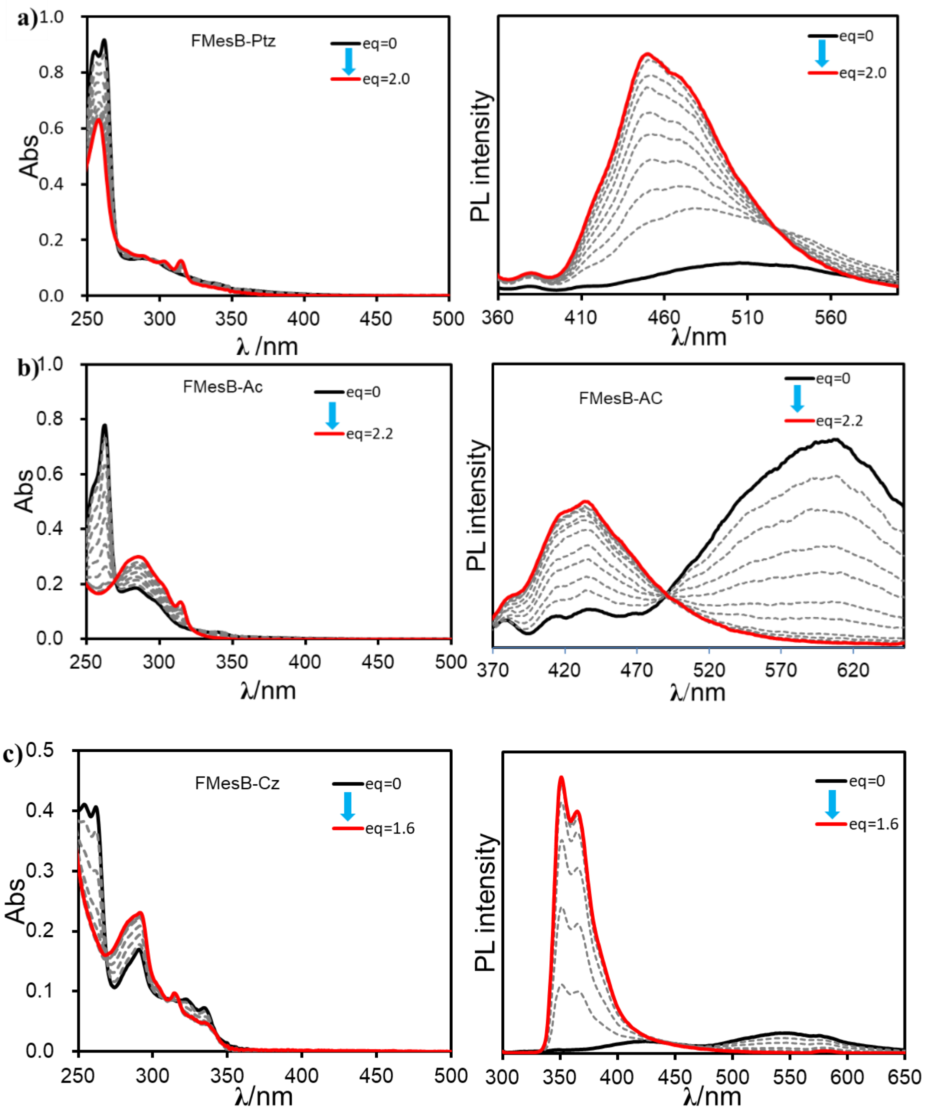


**Figure S7**. UV-Vis absorption spectra (left) and PL spectra (right) of (a) **FMesB-AC**, (b) **FMesB-PTZ**, and (c) **FMesB-Cz** in $CH_2Cl_2$ upon addition of TABF.

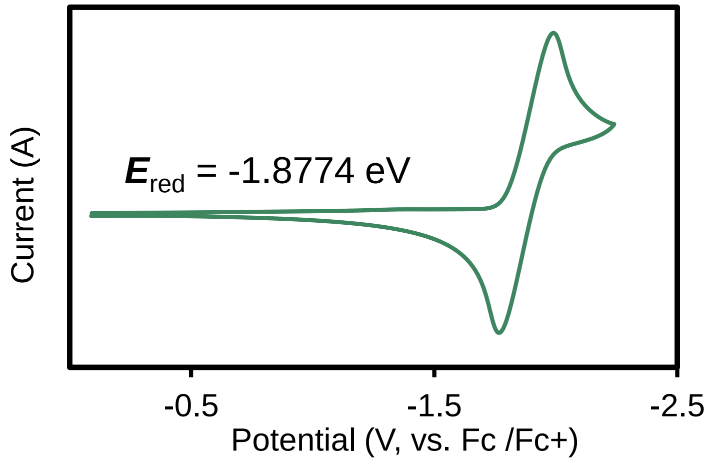


**Figure S8** Cyclic voltammogram of FMes-capped 9-borafluorene compound (**Mes$^F$BF**).[6] Recorded in $CH_2Cl_2$ using $n$-$Bu_4NPF_6$ (0.1 M) as the electrolyte with a scan rate of 100 mV/s.

**Table S2.** Summarized device performances of **FMesB-Cz**, and **FMesB-Ac**.

| **Devices** | **$V_{on}$ (V)** | **$\lambda_{EL}$ (nm)** | **$L_{max}$(cd m$^{-2}$)** | **$\eta_c$(cd A$^{-1}$)** | **$\eta_p$(lm W$^{-1}$)** | **$\eta_{ext}$(%)** |
|---|---|---|---|---|---|---|
| **FMesB-Cz** | 3.8 | 552 | 22710 @ 11 V | 6.6 | 2.9 | 2.1 |
| **FMesB-Ac** | 3.6 | 552 | 22410 @ 10 V | 7.5 | 3.9 | 2.4 |

Abbreviations: $V_{on}$, voltage required for 1 cd m$^{-2}$; $\lambda_{EL}$, emission maxima; $L_{max}$, maximum luminance and required driving voltage; $\eta_c$, maximum current efficiency; $\eta_p$, maximum power efficiency; $\eta_{ext}$, maximum external quantum efficiency.

## S5. DFT Calculations

**Table S3.** Coordinates of **FMesB-Cz** optimised using Gaussian 09 (B3LYP/6-31G**).

| Elements | Coordinates | | | Elements | Coordinates | | |
|---|---|---|---|---|---|---|---|
| | X | Y | Z | | X | Y | Z |
| C | 0.0664 | 0.75873 | -0.88154 | C | -0.27759 | -6.8497 | -0.54637 |
| C | -0.63884 | -0.43893 | -0.85756 | C | 0.76823 | -5.9414 | -0.65078 |
| C | -0.56222 | -1.34257 | 0.2183 | C | 0.53949 | -4.58289 | -0.41569 |
| C | 0.25952 | -0.93841 | 1.2863 | C | -1.52659 | -0.72647 | -2.04624 |
| C | 0.94878 | 0.26818 | 1.28486 | F | -1.66469 | -2.04808 | -2.27445 |
| C | 0.86942 | 1.12513 | 0.19295 | F | -2.77115 | -0.22556 | -1.86986 |
| N | 1.59149 | 2.33709 | 0.17705 | F | -1.04891 | -0.17927 | -3.18086 |
| B | -1.3294 | -2.72689 | 0.21715 | C | 0.45452 | -1.82526 | 2.49411 |
| C | 1.05912 | 3.60789 | -0.06691 | F | 1.52671 | -2.63741 | 2.34757 |
| C | 2.10042 | 4.56014 | 0.00419 | F | 0.6651 | -1.11259 | 3.61843 |
| C | 3.31521 | 3.83555 | 0.30108 | F | -0.61229 | -2.61732 | 2.72342 |
| C | 2.9666 | 2.47024 | 0.40137 | H | 0.00562 | 1.40355 | -1.74658 |
| C | 4.64538 | 4.2198 | 0.47106 | H | 1.54384 | 0.55083 | 2.14144 |
| C | 5.60202 | 3.24952 | 0.72835 | H | 4.92907 | 5.26251 | 0.39804 |
| C | 5.24271 | 1.89903 | 0.80755 | H | 6.63702 | 3.53538 | 0.86443 |
| C | 3.92591 | 1.4901 | 0.64395 | H | 6.00538 | 1.15475 | 0.99878 |
| C | -0.25888 | 3.98751 | -0.3077 | H | 3.66591 | 0.44186 | 0.69819 |
| C | -0.51891 | 5.33781 | -0.50035 | H | -1.06156 | 3.26347 | -0.33845 |
| C | 0.50382 | 6.29215 | -0.44915 | H | -1.53593 | 5.65627 | -0.69128 |
| C | 1.81203 | 5.91057 | -0.19251 | H | 0.26758 | 7.33675 | -0.60553 |
| C | -2.84314 | -2.99636 | 0.49954 | H | 2.5991 | 6.65282 | -0.14151 |
| C | -3.05616 | -4.39151 | 0.37911 | H | -3.76591 | -1.09995 | 0.92586 |
| C | -1.78994 | -5.07863 | 0.02285 | H | -6.01087 | -2.07503 | 1.29688 |
| C | -0.73301 | -4.14171 | -0.07831 | H | -6.3579 | -4.50563 | 1.08275 |
| C | -3.90873 | -2.1703 | 0.83125 | H | -4.48239 | -6.00273 | 0.49851 |
| C | -5.17778 | -2.71728 | 1.0403 | H | -2.36945 | -7.14854 | -0.13296 |
| C | -5.37212 | -4.08727 | 0.91881 | H | -0.09446 | -7.90164 | -0.72973 |
| C | -4.31106 | -4.93655 | 0.58731 | H | 1.75965 | -6.28827 | -0.91375 |
| C | -1.5667 | -6.42473 | -0.20803 | H | 1.36151 | -3.88074 | -0.49559 |

**Table S4.** Coordinates of **FMesB-Ac** optimised using Gaussian 09 (B3LYP/6-31G**).

| Elements | Coordinates | | | Elements | Coordinates | | |
|---|---|---|---|---|---|---|---|
| | X | Y | Z | | X | Y | Z |
| C | -0.64124 | -0.417 | -1.12091 | C | 6.75042 | 0.34198 | 1.21978 |
| C | 0.75446 | -0.41 | -1.12913 | C | -1.34536 | -0.93304 | -2.35182 |
| C | 1.46192 | 0 | -1.79E-06 | F | -1.62784 | -2.25217 | -2.24754 |
| C | 0.75445 | 0.41 | 1.12913 | F | -2.52082 | -0.29294 | -2.56225 |
| C | -0.64124 | 0.417 | 1.12091 | F | -0.60094 | -0.77717 | -3.46612 |
| C | -1.39004 | 0 | -4.15E-06 | C | -1.34537 | 0.93301 | 2.35181 |
| N | 2.89279 | 0 | 4.10E-07 | F | -1.6279 | 2.25212 | 2.2475 |
| C | -3.92934 | 1.234 | -0.08773 | F | -2.52081 | 0.29286 | 2.56227 |
| C | -5.26211 | 0.742 | -0.05743 | F | -0.60094 | 0.77719 | 3.4661 |
| C | -5.26212 | -0.742 | 0.05742 | H | 1.29702 | -0.72298 | -2.01358 |

| | | | | | | | |
|---|---|---|---|---|---|---|---|
| C | -3.92937 | -1.234 | 0.08772 | H | 1.29701 | 0.72297 | 2.01358 |
| B | -2.97284 | 0 | -1.17E-05 | H | -2.69554 | 2.99837 | -0.20323 |
| C | -3.70846 | 2.606 | -0.19155 | H | -4.62861 | 4.55604 | -0.34828 |
| C | -4.79701 | 3.486 | -0.26812 | H | -6.93866 | 3.67574 | -0.29787 |
| C | -6.09912 | 2.989 | -0.2386 | H | -7.36477 | 1.24126 | -0.10901 |
| C | -6.34301 | 1.61 | -0.13221 | H | -7.3648 | -1.24117 | 0.10901 |
| C | -6.34305 | -1.61 | 0.13221 | H | -6.93875 | -3.67566 | 0.29789 |
| C | -6.09919 | -2.989 | 0.23861 | H | -4.62872 | -4.55602 | 0.3483 |
| C | -4.79709 | -3.486 | 0.26814 | H | -2.69561 | -2.99839 | 0.20324 |
| C | -3.70852 | -2.606 | 0.19156 | H | 6.69593 | -2.472 | 0.68696 |
| C | 3.57911 | 1.184 | -0.3407 | H | 5.42011 | -4.49111 | 1.273 |
| C | 4.9884 | 1.22 | -0.34365 | H | 2.91773 | -4.39788 | 1.27731 |
| C | 5.85225 | 0 | 4.36E-06 | H | 1.77104 | -2.3151 | 0.69204 |
| C | 4.98841 | -1.22 | 0.34366 | H | 1.77102 | 2.31509 | -0.69205 |
| C | 3.57912 | -1.184 | 0.3407 | H | 2.9177 | 4.39788 | -1.2773 |
| C | 5.61118 | -2.428 | 0.68305 | H | 5.42009 | 4.49112 | -1.27299 |
| C | 4.89702 | -3.575 | 1.01687 | H | 6.69592 | 2.47202 | -0.68695 |
| C | 3.50492 | -3.522 | 1.01763 | H | 6.13929 | -0.58788 | -2.09284 |
| C | 2.85315 | -2.341 | 0.68466 | H | 7.39639 | -1.19824 | -1.00566 |
| C | 2.85313 | 2.341 | -0.68466 | H | 7.39481 | 0.50162 | -1.48348 |
| C | 3.5049 | 3.522 | -1.01762 | H | 6.13928 | 0.5879 | 2.09285 |
| C | 4.897 | 3.575 | -1.01687 | H | 7.39638 | 1.19826 | 1.00568 |
| C | 5.61116 | 2.428 | -0.68304 | H | 7.39481 | -0.50159 | 1.48349 |
| C | 6.75043 | -0.342 | -1.21977 | | | | |

**Table S5.** Coordinates of **FMesB-Ptz** optimised using Gaussian 09 (B3LYP/6-31G**).

| Elements | Coordinates | | | Elements | Coordinates | | |
|---|---|---|---|---|---|---|---|
| | X | Y | Z | | X | Y | Z |
| C | -0.41845 | 0.415 | 1.14832 | C | 3.86536 | -3.65532 | 0.29508 |
| C | 0.97708 | 0.404 | 1.1483 | C | 5.21236 | -3.63497 | 0.6472 |
| C | 1.67833 | -0.006 | 0.01605 | C | 5.85472 | -2.40696 | 0.80346 |
| C | 0.96148 | -0.406 | -1.1126 | C | -1.11413 | 0.92598 | 2.38647 |
| C | -0.4344 | -0.407 | -1.09693 | F | -1.39365 | 2.24614 | 2.29089 |
| C | -1.17545 | 0.006 | 0.03047 | F | -2.29034 | 0.2874 | 2.5985 |
| N | 3.11145 | 0.005 | 0.04188 | F | -0.36446 | 0.76105 | 3.49524 |
| C | -3.71658 | -1.224 | 0.12295 | C | -1.14515 | -0.91417 | -2.32788 |
| C | -5.04853 | -0.728 | 0.09867 | F | -1.42479 | -2.23466 | -2.23249 |
| C | -5.04603 | 0.755 | -0.0055 | F | -2.32133 | -0.27387 | -2.5285 |
| C | -3.7124 | 1.246 | -0.03588 | F | -0.40529 | -0.74925 | -3.44476 |
| B | -2.75837 | 0.009 | 0.04031 | H | 1.52876 | 0.71142 | 2.02904 |
| C | -3.4979 | -2.596 | 0.2171 | H | 1.49294 | -0.71467 | -2.00569 |
| C | -4.58786 | -3.475 | 0.2895 | H | -2.48565 | -2.99078 | 0.22516 |
| C | -5.88912 | -2.975 | 0.26549 | H | -4.42125 | -4.54602 | 0.36236 |
| C | -6.13079 | -1.596 | 0.16915 | H | -6.72977 | -3.66133 | 0.32139 |
| C | -6.12546 | 1.627 | -0.07047 | H | -7.15194 | -1.22489 | 0.15027 |
| C | -5.87923 | 3.005 | -0.16665 | H | -7.14785 | 1.25947 | -0.047 |
| C | -4.57629 | 3.501 | -0.19589 | H | -6.71765 | 3.69453 | -0.21786 |
| C | -3.48914 | 2.618 | -0.1295 | H | -4.4061 | 4.57094 | -0.26759 |
| C | 3.80101 | -1.223 | 0.24366 | H | -2.47559 | 3.00875 | -0.14033 |
| C | 5.1547 | -1.212 | 0.63227 | H | 6.85666 | 2.62437 | -0.4178 |
| S | 5.95409 | 0.329 | 1.03002 | H | 5.67844 | 4.26822 | -1.86907 |
| C | 5.13288 | 1.38 | -0.14892 | H | 3.27307 | 3.89797 | -2.4235 |
| C | 3.7833 | 1.148 | -0.47604 | H | 2.09416 | 1.92358 | -1.57121 |
| C | 5.81049 | 2.483 | -0.67145 | H | 2.12516 | -2.50777 | -0.2015 |
| C | 5.14892 | 3.403 | -1.48459 | H | 3.34475 | -4.60007 | 0.17207 |
| C | 3.80688 | 3.194 | -1.7924 | H | 5.75967 | -4.55913 | 0.80187 |
| C | 3.13395 | 2.072 | -1.30814 | H | 6.90336 | -2.36568 | 1.08194 |
| C | 3.16939 | -2.465 | 0.08081 | | | | |

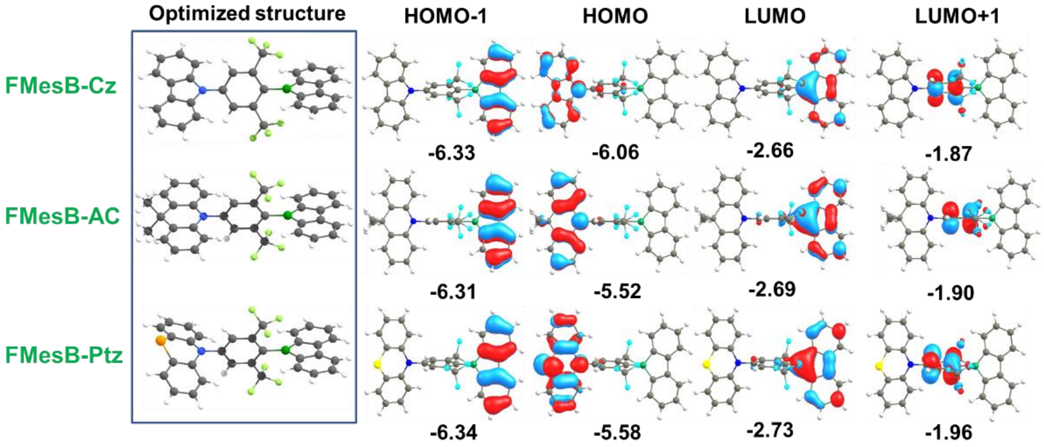


**Figure S9.** Plots of frontier orbitals of donor-functionalized borafluorenes. Geometry optimisations were conducted using Gaussian 09 D.01 at the B3LYP/6-31G** level of theory, and the energy levels were obtained by single point energy calculations at the B3PW91/6-311+G* level of theory.

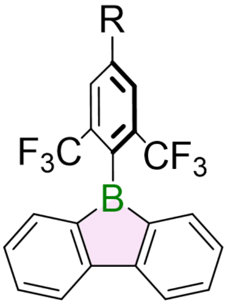


| Compounds | NICS(1)_zz |
|---|---|
| FmesB-Cz | 25.75 |
| FmesB-Ac | 26.25 |
| FmesB-Ptz | 26.21 |

**Figure S10.** NICS(1)_zz value of borole ring in the donor-functionalised borafluorene compounds.

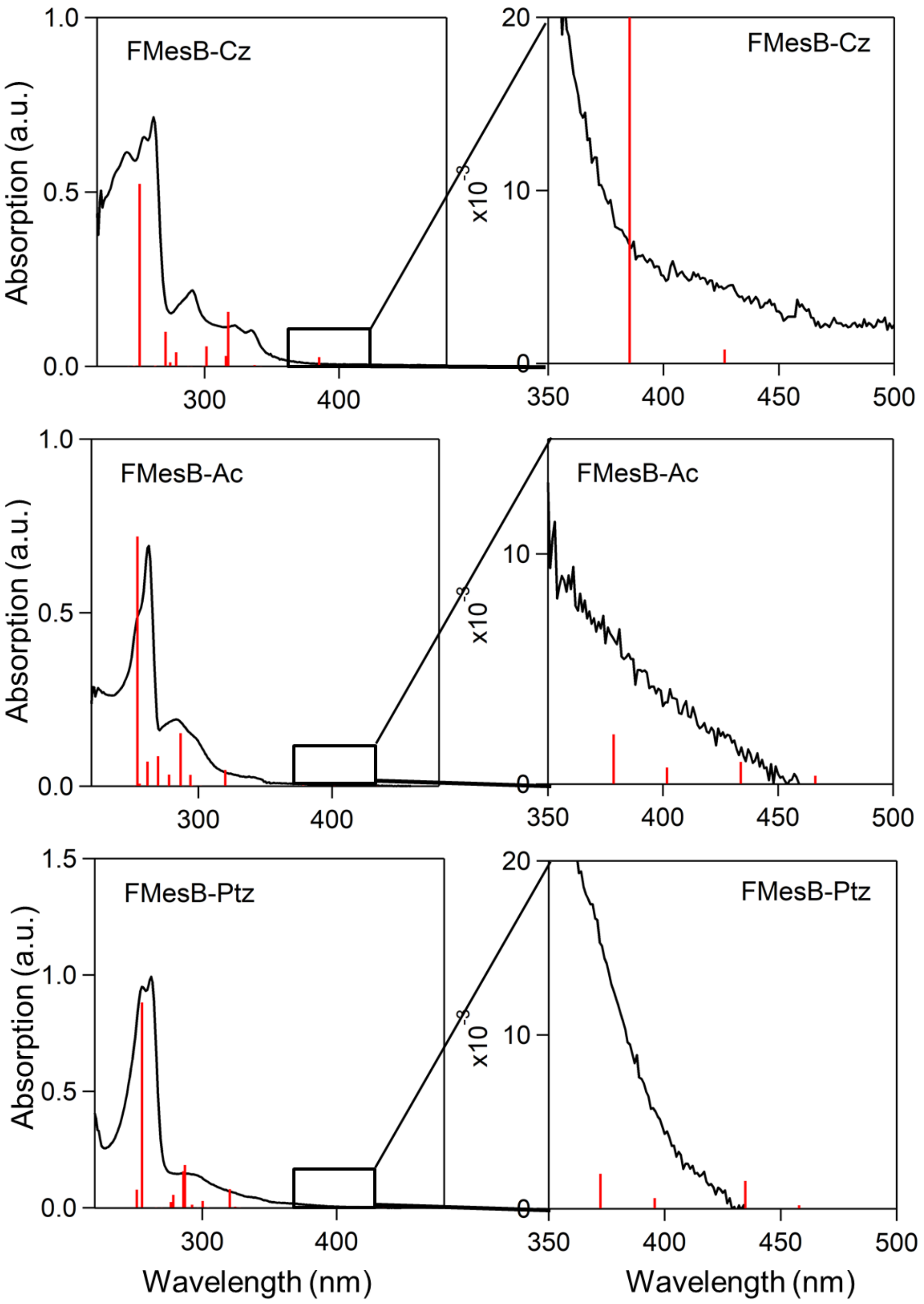


**Figure S11.** UV-Vis spectra of **FMesB-Cz, FMesB-Ac,** and **FMesB-Ptz** in dichloromethane solutions ($1\times10^{-5}$ M), and TD-DFT data (red droplines).

**Table S6.** TD-DFT Data for donor-functionalised borafluorene compounds (PBE0/6-311+G**)

| **FMesB-Cz** | | | | | | | |
|---|---|---|---|---|---|---|---|
| λ(nm) | Oscillator strength f | Assignment | possibility (%) | λ(nm) | Oscillator strength f | Assignment | possibility (%) |
| 426.58 | f=0.0010 | H-1→L | 0.99 | 278.95 | f=0.0504 | H-6→L | 0.44 |
| 385.34 | f=0.0325 | H→L | 0.98 | | | H-5→L | 0.44 |
| 337.19 | f=0.0044 | H→L+1 | 0.99 | 274.58 | f=0.0144 | H-6→L | 0.42 |

| | | | | | | | |
|---|---|---|---|---|---|---|---|
| 317.7 | f=0.1952 | H→L+2 | 0.97 | | | H-5→L | 0.44 |
| 316 | f=0.0372 | H-3→L | 0.78 | 270.94 | f=0.1234 | H-2→L+3 | 0.76 |
| | | H-1→L+4 | 0.2 | | | H→L+5 | 0.17 |
| 301.41 | f=0.0717 | H-2→L+5 | 0.06 | 263.49 | f=0.0024 | H-4→L | 0.97 |
| | | H→L+3 | 0.92 | 251.71 | f=0.6539 | H-3→L | 0.19 |
| 298.92 | f=0.0007 | H-1→L+1 | 1 | | | H-3→L+2 | 0.08 |
| 287.87 | f=0.0010 | H-2→L+2 | 0.97 | | | H-1→L+4 | 0.7 |
| | | | | 247.73 | f=0.0026 | H-3→L+1 | 0.98 |

**FMesB-Ac**

| λ(nm) | Oscillator strength f | Assignment | possibility (%) | λ(nm) | Oscillator strength f | Assignment | possibility (%) |
|---|---|---|---|---|---|---|---|
| 466.22 | f=0.0003 | H→L | 0.96 | 286.78 | f=0.0896 | H-7→L | 0.5 |
| 433.88 | f=0.0008 | H-1→L | 0.99 | | | H-6→L | 0.43 |
| 401.76 | f=0.0006 | H→L+1 | 0.99 | 278.15 | f=0.0275 | H-7→L | 0.41 |
| 378.6 | f=0.0018 | H→L+2 | 0.95 | | | H-6→L | 0.46 |
| 320.24 | f=0.0388 | H-4→L | 0.79 | 269.97 | f=0.0718 | H-2→L+1 | 0.12 |
| | | H-1→L+3 | 0.19 | | | H→L+6 | 0.78 |
| 301.81 | f=0.0010 | H-1→L+1 | 1 | 262.01 | f=0.0591 | H-2→L+1 | 0.86 |
| 297.31 | f=0.0003 | H-2→L | 0.98 | 256.2 | f=0.0057 | H-3→L+1 | 0.99 |
| 295.32 | f=0.0015 | H→L+3 | 0.95 | 254.57 | f=0.5991 | H-4→L | 0.17 |
| 294.17 | f=0.0269 | H→L+4 | 0.89 | | | H-4→L+2 | 0.15 |
| 286.81 | f=0.1268 | H→L+5 | 0.93 | | | H→L+3 | 0.64 |

**FMesB-Ptz**

| λ(nm) | Oscillator strength f | Assignment | possibility (%) | λ(nm) | Oscillator strength f | Assignment | possibility (%) |
|---|---|---|---|---|---|---|---|
| 457.95 | f=0.0001 | H→L | 0.97 | 287.1 | f=0.0916 | H-7→L | 0.42 |
| 434.78 | f=0.0008 | H-1→L | 0.99 | | | H-6→L | 0.26 |
| 372.3 | f=0.0010 | H→L+2 | 0.94 | | | H-5→L | 0.18 |
| 324.6 | f=0.0021 | H→L+4 | 0.87 | 286.13 | f=0.0785 | H-2→L+1 | 0.38 |
| 320.58 | f=0.0398 | H-3→L | 0.79 | | | H→L+6 | 0.51 |
| | | H-1→L+3 | 0.19 | 278.48 | f=0.0278 | H-7→L | 0.25 |
| 304.19 | f=0.0009 | H-1→L+1 | 0.98 | | | H-6→L | 0.59 |
| 300.3 | f=0.0141 | H→L+5 | 0.94 | 276.77 | f=0.0120 | H-2→L+2 | 0.93 |
| 292.44 | f=0.0065 | H-2→L+1 | 0.59 | 255.26 | f=0.4405 | H-3→L | 0.15 |
| | | H→L+6 | 0.36 | | | H-3→L+2 | 0.17 |
| | | | | | | H-1→L+4 | 0.57 |
| | | | | 251.39 | f=0.0387 | H-3→L+1 | 0.89 |

**Table S7.** Coordinates of optimised first excited states ($S_1$) using Gaussian 09 (PBE0/6-31G**).

**FMesB-Cz**

| Elements | Coordinates | | | Elements | Coordinates | | |
|---|---|---|---|---|---|---|---|
| | X | Y | Z | | X | Y | Z |
| C | -1.19801 | -0.85907 | -0.83661 | C | 5.97733 | -1.36235 | 0.81066 |
| C | 0.19335 | -0.86109 | -0.82193 | C | 5.97583 | 1.3642 | -0.8125 |
| C | 0.95137 | -5.80E-04 | 4.50E-04 | C | 5.74387 | 2.52998 | -1.50341 |
| C | 0.19357 | 0.86 | 0.82292 | C | 4.41192 | 2.96469 | -1.74935 |
| C | -1.19782 | 0.85831 | 0.83759 | C | 3.31356 | 2.24341 | -1.30567 |
| C | -1.90401 | -2.60E-04 | 4.70E-04 | C | 0.88676 | -1.82685 | -1.74925 |
| N | -3.31388 | -5.00E-05 | 4.20E-04 | F | 1.84129 | -1.23524 | -2.48679 |
| B | 2.53319 | -3.70E-04 | 1.20E-04 | F | 1.47658 | -2.83376 | -1.07814 |
| C | -4.12694 | -1.11651 | 0.1775 | F | 0.02976 | -2.39637 | -2.61537 |
| C | -5.48033 | -0.71264 | 0.11393 | C | 0.88708 | 1.82581 | 1.75013 |
| C | -5.48009 | 0.71308 | -0.11436 | F | 1.47521 | 2.83372 | 1.07895 |

| | | | | | | | |
|---|---|---|---|---|---|---|---|
| C | -4.12657 | 1.11661 | -0.17717 | F | 0.03043 | 2.394 | 2.61748 |
| C | -6.49218 | 1.66166 | -0.28018 | F | 1.84295 | 1.23475 | 2.4863 |
| C | -6.14306 | 2.98563 | -0.51012 | H | -1.73724 | -1.51446 | -1.51056 |
| C | -4.79502 | 3.36617 | -0.58369 | H | -1.7369 | 1.51376 | 1.51159 |
| C | -3.76923 | 2.44243 | -0.42261 | H | -7.53625 | 1.3649 | -0.23408 |
| C | -3.77006 | -2.44241 | 0.42314 | H | -6.91857 | 3.7344 | -0.63907 |
| C | -4.79617 | -3.3659 | 0.58365 | H | -4.54311 | 4.40537 | -0.77366 |
| C | -6.14407 | -2.98503 | 0.50933 | H | -2.73012 | 2.74657 | -0.49168 |
| C | -6.49275 | -1.66098 | 0.27919 | H | -2.73106 | -2.7468 | 0.49279 |
| C | 3.49167 | -1.04809 | 0.59595 | H | -4.54462 | -4.40516 | 0.77376 |
| C | 4.87047 | -0.60886 | 0.3542 | H | -6.91983 | -3.73361 | 0.63785 |
| C | 4.86981 | 0.60987 | -0.3554 | H | -7.53671 | -1.36396 | 0.23252 |
| C | 3.49054 | 1.0481 | -0.59626 | H | 2.31549 | -2.61389 | 1.50731 |
| C | 3.31603 | -2.24348 | 1.30557 | H | 4.26523 | -3.88828 | 2.29975 |
| C | 4.41519 | -2.9639 | 1.74863 | H | 6.57709 | -3.12431 | 1.86612 |
| C | 5.74667 | -2.52824 | 1.50182 | H | 6.99192 | -1.02391 | 0.62161 |
| H | 4.26093 | 3.88901 | -2.30029 | H | 6.99078 | 1.0265 | -0.6241 |
| H | 2.31262 | 2.6131 | -1.50677 | H | 6.57362 | 3.1267 | -1.86819 |

**FMesB-Ac**

| Elements | Coordinates | | | Elements | Coordinates | | |
|---|---|---|---|---|---|---|---|
| | X | Y | Z | | X | Y | Z |
| C | 0.62542 | -0.4551 | 1.1072 | C | -4.83784 | 3.40883 | 1.39265 |
| C | -0.76346 | -0.45984 | 1.1139 | C | -5.57957 | 2.32024 | 0.94705 |
| C | -1.45229 | 1.00E-05 | -2.00E-05 | C | -6.70996 | -0.47765 | 1.1712 |
| C | -0.7635 | 0.45985 | -1.11396 | C | -6.71 | 0.47765 | -1.17107 |
| C | 0.62538 | 0.45512 | -1.1073 | C | 1.28955 | -0.91903 | 2.38042 |
| C | 1.39409 | 1.00E-05 | -6.00E-05 | F | 1.76135 | -2.17453 | 2.28845 |
| N | -2.89356 | 0 | 0 | F | 2.30942 | -0.13321 | 2.75053 |
| C | 3.91948 | 1.22203 | -0.07445 | F | 0.42003 | -0.92374 | 3.41402 |
| C | 5.2639 | 0.73035 | -0.02311 | C | 1.28946 | 0.91903 | -2.38055 |
| C | 5.26389 | -0.73036 | 0.02317 | F | 1.76127 | 2.17453 | -2.28862 |
| C | 3.91946 | -1.22203 | 0.07442 | F | 2.30933 | 0.13321 | -2.7507 |
| B | 2.96612 | 1.00E-05 | -8.00E-05 | F | 0.4199 | 0.92372 | -3.41412 |
| C | 3.73618 | 2.61446 | -0.07845 | H | -1.31037 | -0.79653 | 1.9872 |
| C | 4.82498 | 3.48081 | -0.05862 | H | -1.31043 | 0.79653 | -1.98725 |
| C | 6.13325 | 2.97808 | -0.02454 | H | 2.73049 | 3.02723 | -0.10604 |
| C | 6.35172 | 1.60118 | -0.00228 | H | 4.66345 | 4.55622 | -0.06823 |
| C | 6.35171 | -1.6012 | 0.00244 | H | 6.97745 | 3.66234 | -0.00709 |
| C | 6.13322 | -2.9781 | 0.02471 | H | 7.36877 | 1.21541 | 0.03747 |
| C | 4.82494 | -3.48081 | 0.05871 | H | 7.36876 | -1.21544 | -0.03725 |
| C | 3.73615 | -2.61446 | 0.07844 | H | 6.97741 | -3.66237 | 0.00734 |
| C | -3.55653 | 1.12389 | 0.45966 | H | 4.6634 | -4.55622 | 0.06833 |
| C | -4.97221 | 1.16114 | 0.474 | H | 2.73046 | -3.02722 | 0.10596 |
| C | -5.81949 | 0 | 5.00E-05 | H | -6.66224 | -2.38119 | -0.97121 |
| C | -4.97222 | -1.16114 | -0.47392 | H | -5.34828 | -4.29496 | -1.75406 |
| C | -3.55654 | -1.12388 | -0.45963 | H | -2.85634 | -4.20413 | -1.72183 |
| C | -5.5796 | -2.32024 | -0.94695 | H | -1.72093 | -2.18489 | -0.8981 |
| C | -4.83787 | -3.40883 | -1.39258 | H | -1.72091 | 2.18491 | 0.89807 |
| C | -3.44081 | -3.35951 | -1.37492 | H | -2.8563 | 4.20414 | 1.72185 |
| C | -2.80161 | -2.23017 | -0.91394 | H | -5.34824 | 4.29496 | 1.75415 |
| C | -2.80159 | 2.23018 | 0.91395 | H | -6.66222 | 2.38118 | 0.97134 |
| C | -3.44078 | 3.35952 | 1.37495 | H | -6.09958 | -0.82248 | 2.00973 |
| H | -7.35531 | 1.29915 | -0.85113 | H | -7.35528 | -1.29915 | 0.85128 |
| H | -7.35144 | -0.3349 | -1.52009 | H | -7.35139 | 0.3349 | 1.52024 |
| H | -6.09964 | 0.82248 | -2.00962 | | | | |

| FMesB-Ptz | | | | | | | |
|---|---|---|---|---|---|---|---|
| Elements | Coordinates | | | Elements | Coordinates | | |
| | X | Y | Z | | X | Y | Z |
| C | -0.41936 | -0.2005 | -1.24427 | C | 3.04785 | -2.15941 | 1.0811 |
| C | 0.97293 | -0.22135 | -1.26324 | C | 3.18277 | 2.43319 | 0.01494 |
| C | 1.68514 | 0.02605 | -0.10044 | C | 3.89592 | 3.62519 | -0.06538 |
| C | 0.99829 | 0.30444 | 1.07573 | C | 5.25878 | 3.61975 | -0.33711 |
| C | -0.39204 | 0.31597 | 1.08304 | C | 5.90171 | 2.40486 | -0.5505 |
| C | -1.15426 | 0.05792 | -0.07291 | C | -1.14001 | -0.48662 | -2.54113 |
| N | 3.11381 | -0.02167 | -0.13792 | F | -1.74465 | -1.68964 | -2.52867 |
| C | -3.73165 | 1.18676 | -0.00659 | F | -2.09004 | 0.42624 | -2.81056 |
| C | -5.09087 | 0.63389 | 0.01626 | F | -0.30089 | -0.4901 | -3.59237 |
| C | -5.03631 | -0.77201 | 0.00182 | C | -1.08263 | 0.62095 | 2.39169 |
| C | -3.63885 | -1.21495 | -0.04586 | F | -1.71382 | 1.80959 | 2.36265 |
| B | -2.7387 | 0.02298 | -0.04734 | F | -2.00366 | -0.3048 | 2.7128 |
| C | -3.60127 | 2.58652 | -0.00537 | F | -0.21522 | 0.6724 | 3.41882 |
| C | -4.7243 | 3.38875 | 0.01691 | H | 1.51467 | -0.42841 | -2.17785 |
| C | -6.04043 | 2.83774 | 0.04288 | H | 1.55557 | 0.49895 | 1.98448 |
| C | -6.22796 | 1.47943 | 0.04337 | H | -2.61452 | 3.0379 | -0.01833 |
| C | -6.10196 | -1.70593 | 0.0303 | H | -4.61513 | 4.46891 | 0.01605 |
| C | -5.80669 | -3.04494 | 0.01563 | H | -6.89263 | 3.50827 | 0.06088 |
| C | -4.45113 | -3.48906 | -0.02938 | H | -7.22809 | 1.05821 | 0.06248 |
| C | -3.39662 | -2.59947 | -0.06061 | H | -7.13239 | -1.36687 | 0.06557 |
| C | 3.81717 | 1.20564 | -0.20359 | H | -6.6023 | -3.78143 | 0.03829 |
| C | 5.18608 | 1.212 | -0.51206 | H | -4.25729 | -4.55721 | -0.04023 |
| S | 5.98748 | -0.3012 | -0.97319 | H | -2.3772 | -2.96973 | -0.10283 |
| C | 5.10921 | -1.40817 | 0.09807 | H | 6.81653 | -2.66393 | 0.40612 |
| C | 3.74669 | -1.19016 | 0.35324 | H | 5.55627 | -4.37406 | 1.6942 |
| C | 5.75691 | -2.53256 | 0.60112 | H | 3.12121 | -4.03858 | 2.1001 |
| C | 5.05033 | -3.49089 | 1.32049 | H | 1.99105 | -2.03287 | 1.27804 |
| C | 3.69258 | -3.30104 | 1.54638 | H | 2.12323 | 2.46467 | 0.23567 |
| H | 5.81706 | 4.54788 | -0.38813 | H | 3.37522 | 4.56287 | 0.09773 |
| H | 6.96428 | 2.37622 | -0.77036 | | | | |

## S6. NMR Spectra and HRMS

$^{1}$H NMR spectrum of **1** (400 MHz, $CDCl_3$).

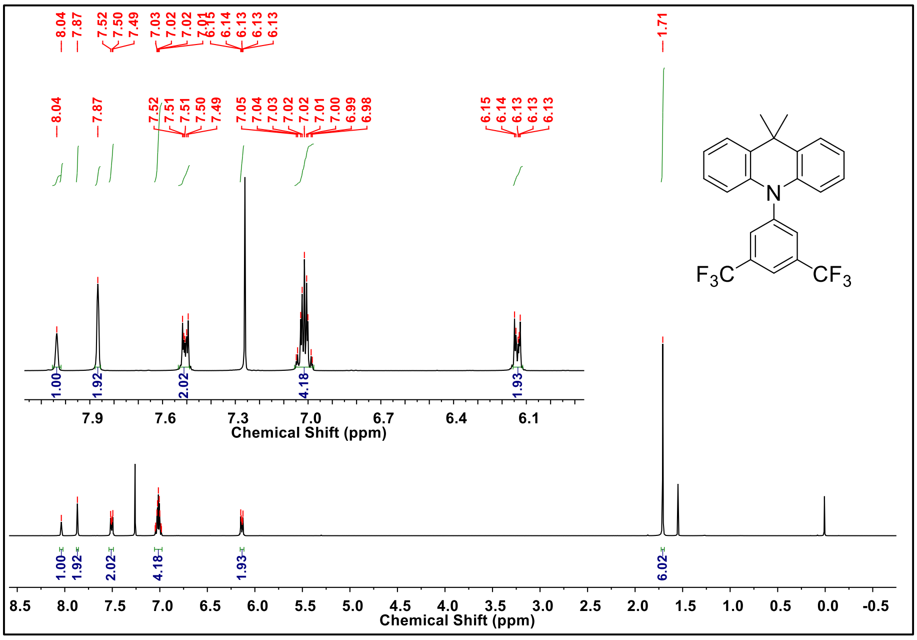


$^{13}$C NMR spectrum of **1** (101 MHz, $CDCl_3$).

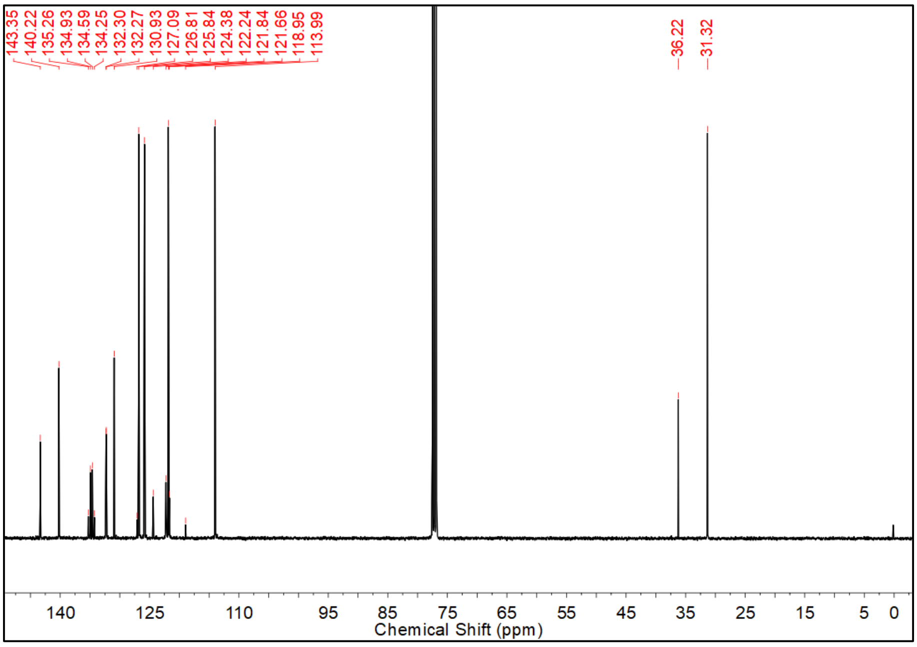


HRMS of compound **1**.

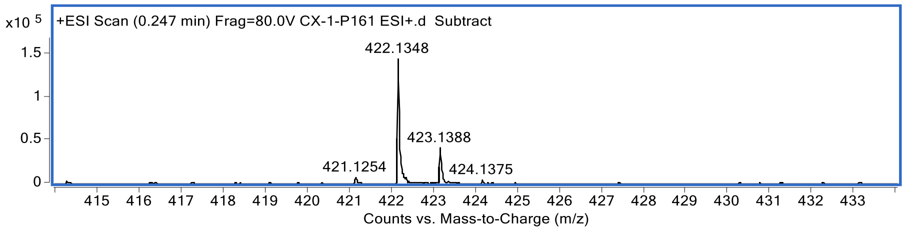

$^{1}$H NMR spectrum of **FMesB-Ac** (400 MHz, $CDCl_3$).

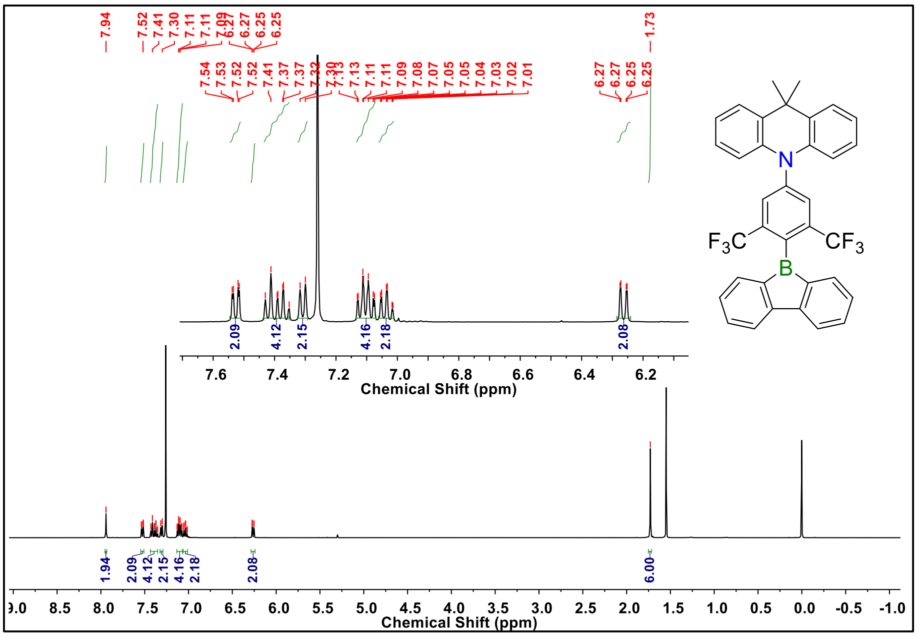

$^{13}$C{$^{1}$H} NMR spectrum of **FMesB-Ac** (101 MHz, $CDCl_3$).

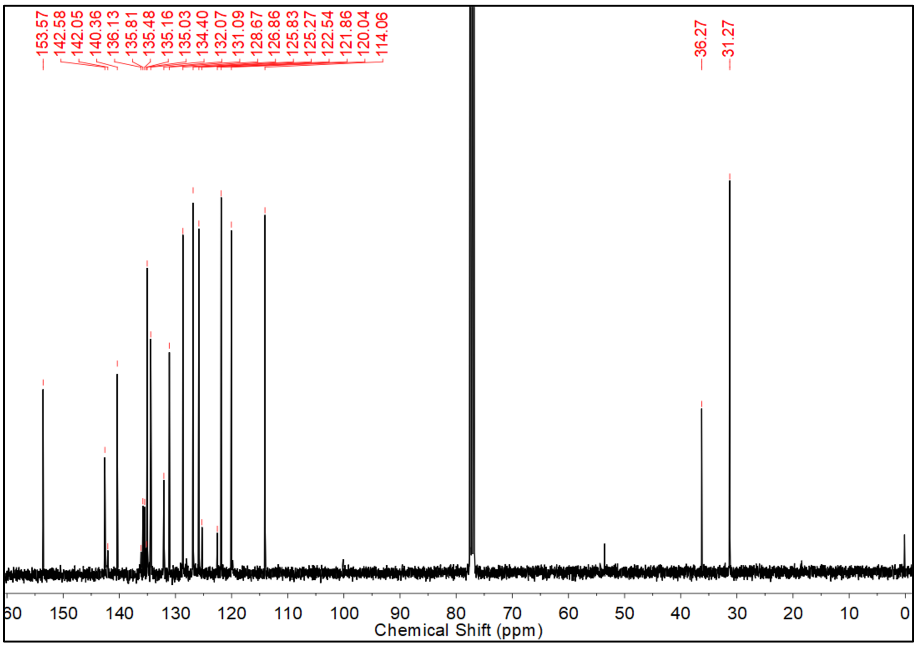

$^{11}$B NMR spectrum of **FMesB-Ac** (128 MHz, $CDCl_3$).

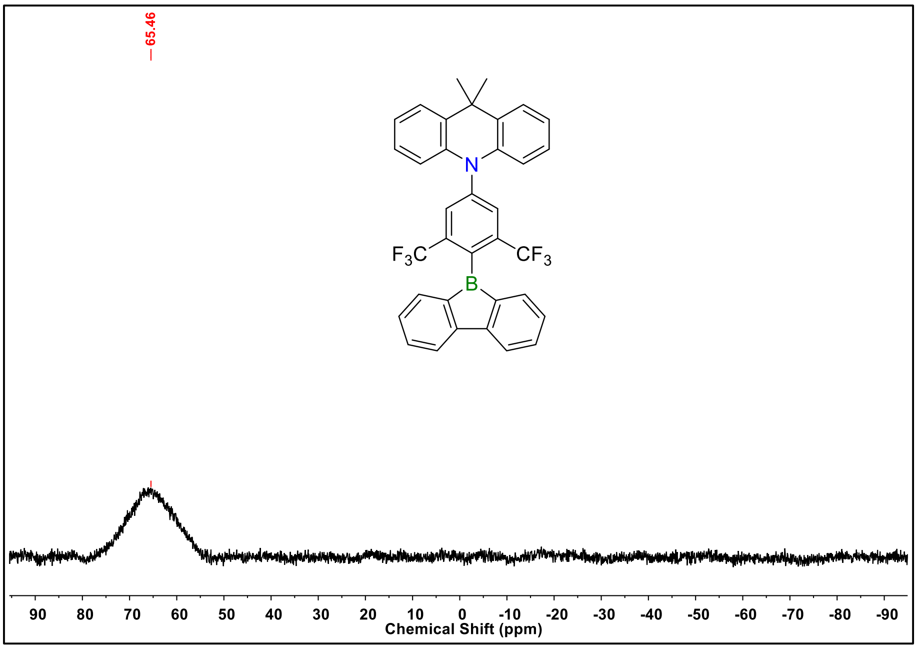


HRMS of compound **FMesB-Ac**.

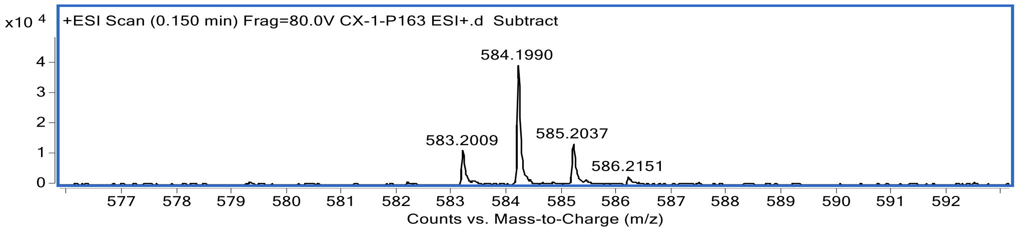


$^1$H NMR spectrum of **FMesB-PTZ** (400 MHz, $CDCl_3$).

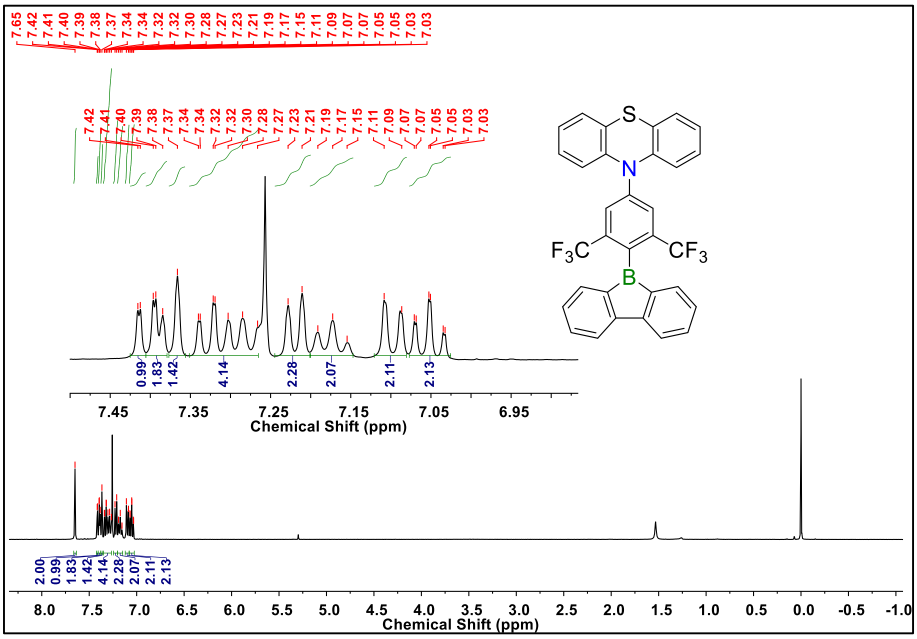

$^{13}C\{^{1}H\}$ NMR spectrum of **FMesB-PTZ** (101 MHz, $CDCl_3$).

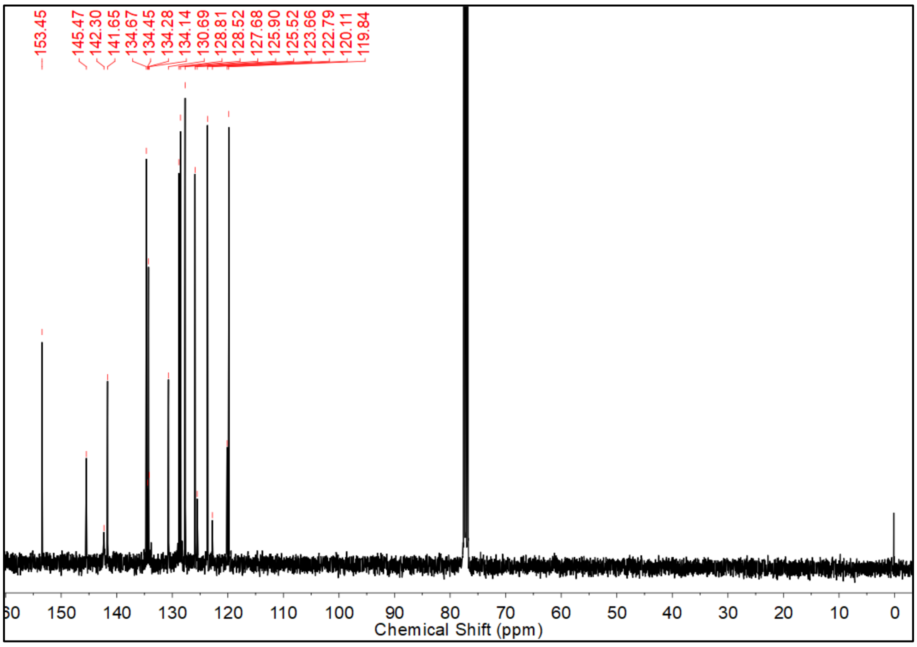


$^{11}B$ NMR spectrum of **FMesB-PTZ** (128 MHz, $CDCl_3$).

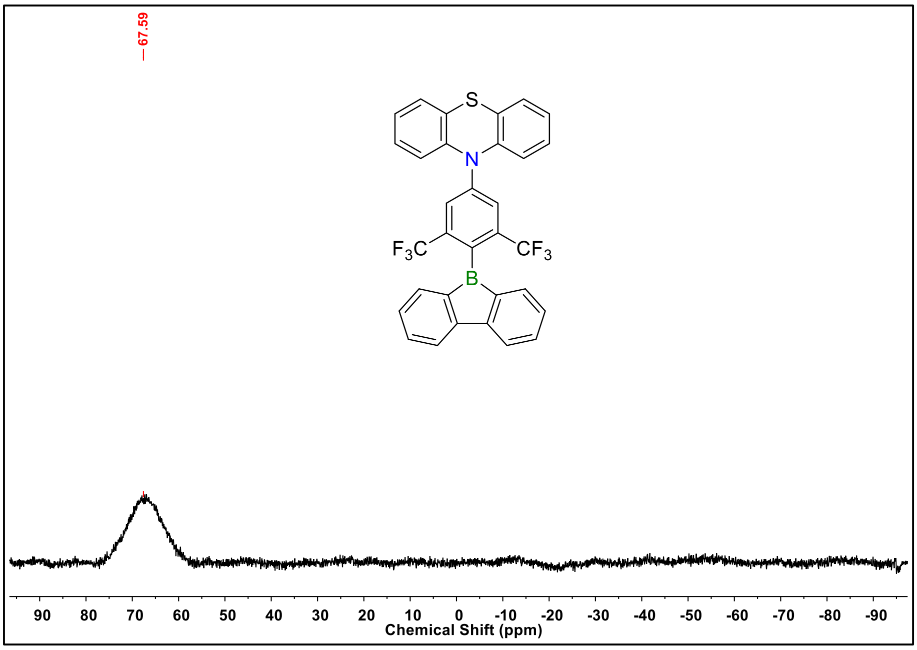


HRMS of compound **FMesB-PTZ**.

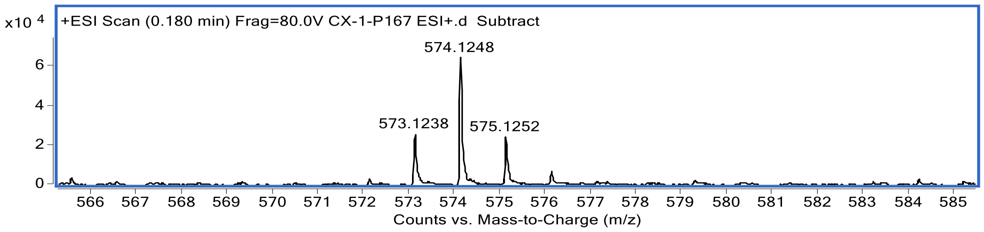

$^{1}H$ NMR spectrum of **FMesB-Cz** (400 MHz, $CDCl_3$).

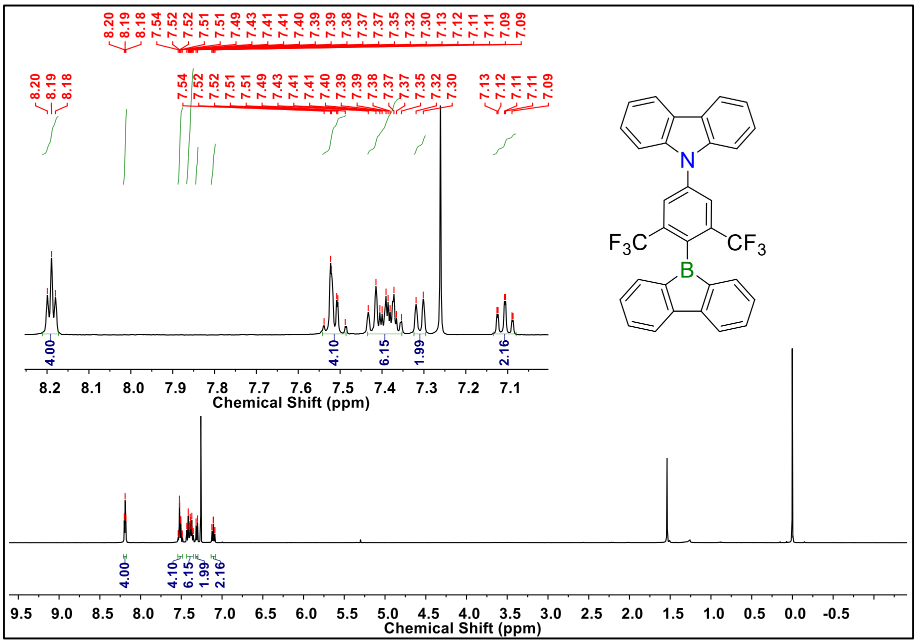


$^{13}C\{^{1}H\}$ NMR spectrum of **FMesB-Cz** (101 MHz, $CDCl_3$).

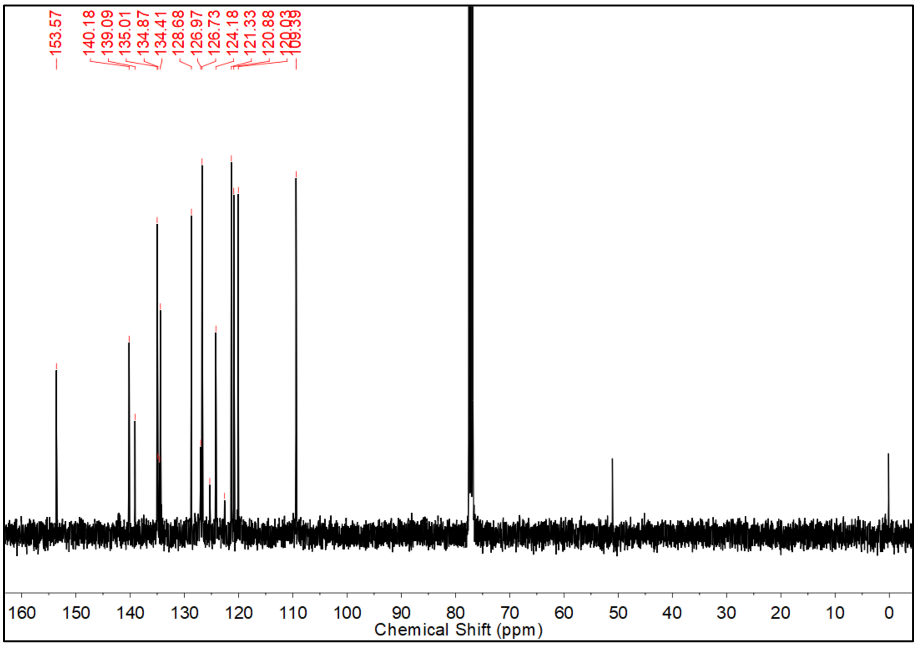

$^{11}B$ NMR spectrum of **FMesB-Cz** (128 MHz, $CDCl_3$).

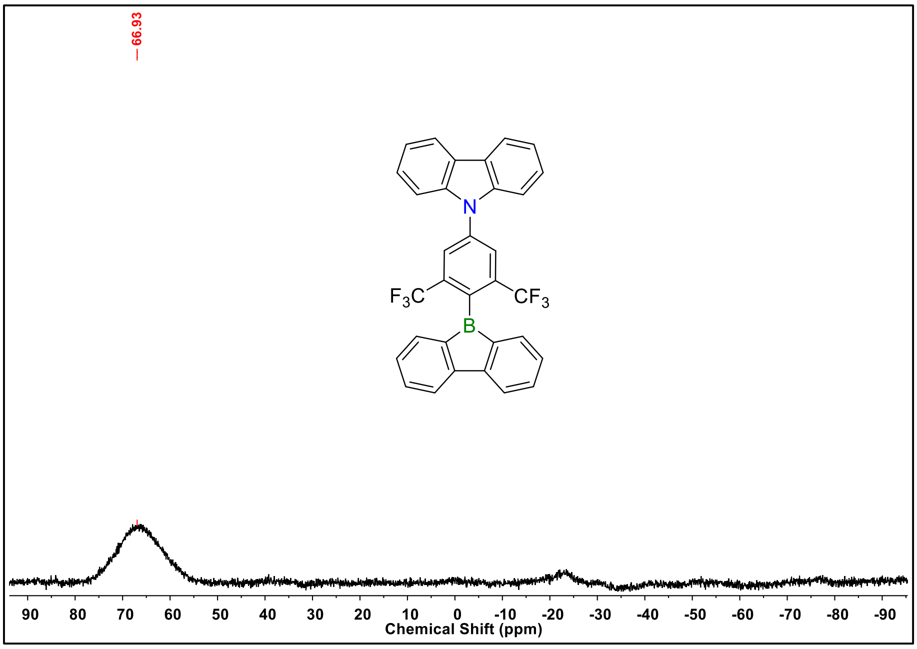


HRMS of compound **FMesB-Cz**.

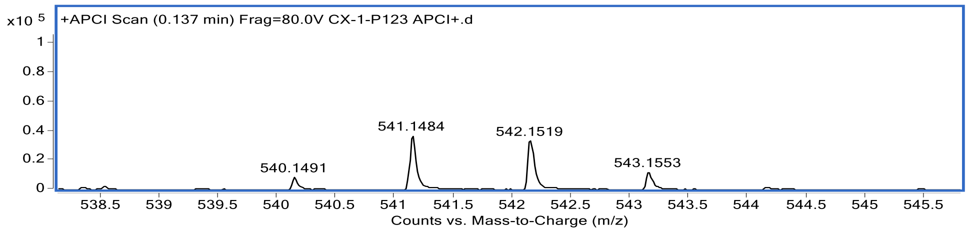


## S7. References


1 M. J. Frisch, G. W. Trucks, H. B. Schlegel, G. E. Scuseria, M. A. Robb, J. R. Cheeseman, G. Scalmani, V. Barone, B. Mennucci, G. A. Petersson, H. Nakatsuji, M. Caricato, X. Li, H. P. Hratchian, A. F. Izmaylov, J. Bloino, G. Zheng, J. L. Sonnenberg, M. Hada, M. Ehara, K. Toyota, R. Fukuda, J. Hasegawa, M. Ishida, T. Nakajima, Y. Honda, O. Kitao, H. Nakai, T. Vreven, J. A. Montgomery Jr., J. E. Peralta, F. Ogliaro, M. J. Bearpark, J. Heyd, E. N. Brothers, K. N. Kudin, V. N. Staroverov, R. Kobayashi, J. Normand, K. Raghavachari, A. P. Rendell, J. C. Burant, S. S. Iyengar, J. Tomasi, M. Cossi, N. Rega, N. J. Millam, M. Klene, J. E. Knox, J. B. Cross, V. Bakken, C. Adamo, J. Jaramillo, R. Gomperts, R. E. Stratmann, O. Yazyev, A. J. Austin, R. Cammi, C. Pomelli, J. W. Ochterski, R. L. Martin, K. Morokuma, V. G. Zakrzewski, G. A. Voth, P. Salvador, J. J. Dannenberg, S. Dapprich, A. D. Daniels, Ö. Farkas, J. B. Foresman, J. V. Ortiz, J. Cioslowski, D. J. Fox, *Gaussian 09, Revision D.01*, 2009.

2 K. T. Kamtekar, K. Dahms, A. S. Batsanov, V. Jankus, H. L. Vaughan, A. P. Monkman, M. R. Bryce, *J. Polym. Sci. Part A Polym. Chem.*, **2011**, *49*, 1129–1137.

3 A. W. Jones, M. Louillat-habermeyer, F. W. Patureau, *Adv.Synth. Catal.*, **2015**, *357*, 945–949.

4 S. Biswas, I. M. Oppel, H. F. Bettinger, *Inorg. Chem.*, **2010**, *49*, 4499–4506.

5 G. P. M. Van Klink, H. J. R. De Boer, G. Schat, O. S. Akkerman, F. Bickelhaupt, A. L. Spek, *Organometallics*, **2002**, *21*, 2119–2135.

6 M. F. Smith, S. J. Cassidy, I. A. Adams, M. Vasiliu, D. L. Gerlach, D. A. Dixon, P. A. Rupar, *Organometallics*, **2016**, *35*, 3182–3191.

7 G. M. Sheldrick, *Acta Crystallogr. Sect. C*, **2015**, *71*, 3–8.

8 G. M. Sheldrick, *Acta Crystallogr. Sect. A*, **2008**, *64*, 112–122.

9 O. V Dolomanov, L. J. Bourhis, R. J. Gildea, J. A. K. Howard, H. Puschmann, *J. Appl. Crystallogr.*, **2009**, *42*, 339–341.
